# Impact of Maternal Underweight on Infant and Maternal Outcomes in Japanese Women: A Systematic Review and Meta-analysis

**DOI:** 10.2188/jea.JE20250483

**Published:** 2026-08-05

**Authors:** Shinobu Kobayashi, Shiori Itoi, Drishti Shrestha, Naho Morisaki

**Affiliations:** 1 Department of Social Medicine, National Center for Child Health and Development, Tokyo, Japan; 2 Division of Reproductive Medicine, National Center for Child Health and Development Research Institute, Tokyo, Japan; 3 Department of Obstetrics and Gynecology, The University of Tokyo, Tokyo, Japan; 4 Graduate School of Public Health, St. Luke’s International University, Tokyo, Japan

**Keywords:** pre-pregnancy BMI, underweight, pregnancy outcomes, Japanese women

## Abstract

**Background:**

Pre-pregnancy underweight (BMI <18.5 kg/m^2^) is notably prevalent among reproductive-aged Japanese women, affecting approximately 20%, compared to less than 10% in Western countries. However, its overall impact on maternal and infant outcomes in Japanese populations has not been systematically evaluated.

**Methods:**

We conducted a systematic review and meta-analysis following Preferred Reporting Items for Systematic Reviews and Meta-Analyses 2020 guidelines. MEDLINE, EMBASE, CINAHL, PsycINFO, Cochrane Library, and *Ichushi* were searched from inception to February 2024. Cohort and case-control studies examining associations between pre-pregnancy underweight and perinatal outcomes in Japanese women with singleton pregnancies were included. Primary outcomes were low birth weight (LBW), small for gestational age (SGA), and preterm birth (PTB). Random-effects models calculated pooled odds ratios (ORs) with 95% confidence intervals (CIs).

**Results:**

Thirty-four studies were analyzed. Pre-pregnancy underweight significantly increased risks of LBW (OR 1.61; 95% CI, 1.38–1.86), SGA (OR 1.59; 95% CI, 1.55–1.63), and PTB (OR 1.23; 95% CI, 1.19–1.26). Mean birth weight was 115.02 g lower (95% CI, −128.05 to −101.99) in underweight mothers.

**Conclusion:**

Pre-pregnancy underweight among Japanese women is significantly associated with increased risks of adverse perinatal outcomes. Notably, these elevated risks persist despite the high background prevalence of underweight, suggesting that their adverse effects are not diminished in populations where it is more common. These findings underscore the importance of increasing awareness of preconception care and emphasize the need to optimize pre-pregnancy weight.

## INTRODUCTION

Pre-pregnancy body mass index (BMI) has been recognized as an important determinant of both maternal and infant health outcomes.^1^^,^^2^ While much attention has been focused on the risks associated with maternal overweight and obesity, pre-pregnancy underweight remains an important yet often overlooked public health issue. Previous studies have linked maternal underweight to adverse perinatal outcomes, such as low birth weight (LBW), small for gestational age (SGA), and preterm birth (PTB).^3^^,^^4^ These outcomes may contribute to elevated risks of neonatal morbidity, mortality, and long-term health consequences, including developmental delays and chronic diseases later in life.^5^^,^^6^

Japan faces a unique public health challenge with its high prevalence of underweight among individuals of reproductive age. National data indicate that approximately 20% in their 20s to 30s are classified as underweight (BMI <18.5 kg/m^2^), which is more than double the prevalence reported in most Western countries.^7^ Despite this distinct demographic pattern, the overall impact of pre-pregnancy underweight on maternal and infant health outcomes in Japanese populations has not been systematically examined.

Although several regional and large-scale cohort studies in Japan have investigated the relationship between pre-pregnancy underweight and various perinatal outcomes,^8^^–^^10^ their findings have not been systematically synthesized. Previous international meta-analyses have included some Asian populations,^3^^,^^11^ but none have specifically focused on the Japanese population. As a result, the strength and consistency of these associations in the Japanese remain unclear.

Therefore, this systematic review and meta-analysis aims to quantitatively assess the impact of pre-pregnancy underweight on maternal and infant outcomes among Japanese individuals. By synthesizing evidence from all available studies conducted in Japanese populations, our findings will help inform clinical practice, public health interventions, and health policies specific to the Japanese context.

## METHODS

This systematic review was conducted according to a protocol registered in PROSPERO (CRD42024524296) and reported following the Preferred Reporting Items for Systematic Reviews and Meta-Analyses (PRISMA) 2020 statement.^12^

### Eligibility criteria

We included studies that met the following criteria: (1) cohort or case-control study design; (2) included Japanese women with singleton pregnancies; (3) reported pre-pregnancy BMI or allowed for the identification of women classified as underweight before pregnancy; (4) included a normal BMI comparison group; (5) reported at least one of our specified maternal or infant outcomes; (6) included a minimum of 200 pregnant women based on the rationale that smaller studies may lack sufficient sample size to detect outcomes across BMI categories; and (7) was published in either English or Japanese. The definition of BMI primarily followed the World Health Organization (WHO) criteria (underweight, BMI <18.5 kg/m^2^; normal BMI, 18.5 ≤ BMI < 25 kg/m^2^).^13^ However, some included studies reported the definition of BMI previously proposed by the Japan Society of Obstetrics and Gynecology (underweight, BMI <18.0 kg/m^2^; normal BMI, 18.0 ≤ BMI < 24 kg/m^2^) and other criteria.^14^

We excluded studies if they: (1) were review articles, case reports, editorials, or conference abstracts; (2) exclusively analyzed non-Japanese populations or did not separate results for Japanese women; (3) exclusively studied multiple pregnancies; or (4) did not provide sufficient data to calculate effect sizes.

### Information sources and search strategy

A comprehensive literature search was conducted in MEDLINE, EMBASE, CINAHL, PsycINFO, and Cochrane Library (CENTRAL) from inception to February 2024. Additionally, we searched *Ichushi*, the Japanese medical database maintained by the Japan Medical Abstracts Society. Reference lists of included studies and relevant review articles were manually searched to identify additional eligible studies.

The search strategy was developed based on the opinions of a medical librarian, controlled vocabularies [CINAHL Heading; Medical Subject Heading (MeSH); Excerpta Medical Tree (EMTREE)], and a review of key search results. The following terms were included: (1) pre-pregnancy or pre-conceptional period; (2) BMI, body weight, or underweight; (3) pregnancy outcomes or perinatal outcomes; and (4) Japanese population (eMaterial 1, eMaterial 2, eMaterial 3, eMaterial 4, eMaterial 5 and eMaterial 6).

### Study selection

After removing duplicates, reviewers (S.I., S.K., and D.S.) independently screened titles and abstracts of all identified articles to determine eligibility. Subsequently, full texts of potentially eligible studies were retrieved and independently assessed by the same two reviewers. Disagreements were resolved through discussion, and when necessary, by consulting a third reviewer (N.M.). The selection process was documented using a PRISMA flow diagram.^12^

### Data collection process

Data extraction was performed independently by reviewers (S.I., S.K., and D.S.) using a pre-piloted standardized form. The following information was extracted from each included study: (1) study characteristics (first author, publication year, study design, study period, sample size); (2) population characteristics (age distribution, parity, socioeconomic factors if available); (3) BMI measurement and categorization methods; (4) outcome definitions and ascertainment methods; (5) adjusted confounding variables; and (6) effect measures (adjusted and unadjusted odds ratios [ORs], risk ratios [RRs], or hazard ratios [HRs] with 95% confidence intervals [CIs]) for the association between pre-pregnancy underweight and outcomes.

### Outcomes

The primary outcomes were: (1) LBW (defined as birth weight <2,500 g); (2) SGA (defined as birth weight less than the 10th percentile for gestational age)^15^; and (3) PTB (defined as birth before 37 weeks of gestation, including both spontaneous and induced).

Secondary infant outcomes included: macrosomia (birth weight ≥4,000 g), large for gestational age (LGA; birth weight ≥90th percentile for gestational age), low Apgar scores, congenital disorders, infant hospitalization, perinatal mortality outcomes, and early childhood growth. Secondary maternal outcomes included: gestational diabetes mellitus, using the definition of the Japanese Society of Obstetrics and Gynecology; hypertensive disorders in pregnancy (HDP), defined as hypertension of systolic blood pressure ≥140 mm Hg and/or diastolic blood pressure ≥90 mm Hg, including preeclampsia, gestational hypertension, superimposed preeclampsia, chronic hypertension; cesarean section, defined as delivery via abdominal hysterotomy (elective or emergency); postpartum hemorrhage, defined as estimated blood loss of 1,000 mL or more for vaginal delivery, and 2,000 mL or more for cesarean section; maternal mortality; and gestational weight gain.

### Quality assessment of included studies

The methodological quality of the included studies was assessed independently by two reviewers (S.I. and S.K.) using the Newcastle-Ottawa Scale (NOS) for cohort and case-control studies.^16^ This scale evaluates studies based on three domains: selection of study groups, comparability of groups, and ascertainment of exposure or outcome. Studies can receive a maximum of 9 stars, with higher scores indicating better methodological quality. Disagreements were resolved through discussion or by consulting a third reviewer (N.M.).

### Statistical analysis

If sufficient comparable data were available, meta-analyses were conducted using a random-effects model to calculate pooled ORs with 95% CIs for dichotomous outcomes. For continuous outcomes, weighted mean differences with 95% CIs were calculated. The main meta-analysis consistently used crude estimates to ensure methodological consistency. When studies reported adjusted effect estimates, results were presented separately.

Statistical heterogeneity among studies was assessed using the I^2^ statistic, with values of 25%, 50%, and 75% considered as low, moderate, and high heterogeneity, respectively.^17^ For primary outcomes with high heterogeneity, a fixed-effects model was also applied as a sensitivity analysis to compare the pooled estimates. For the primary outcomes, the main analysis was restricted to studies using the WHO BMI criteria. A sensitivity analysis was subsequently performed, pooling all eligible studies regardless of the BMI definition used. Publication bias was assessed for the primary outcomes using visual evaluation of funnel plots. Where 10 or more studies were available, Egger’s test was also performed.^18^

All statistical analyses were performed using STATA version 18.0 (Stata Corp, College Station, TX, USA).

## RESULTS

### Study selection

**Figure 1.**
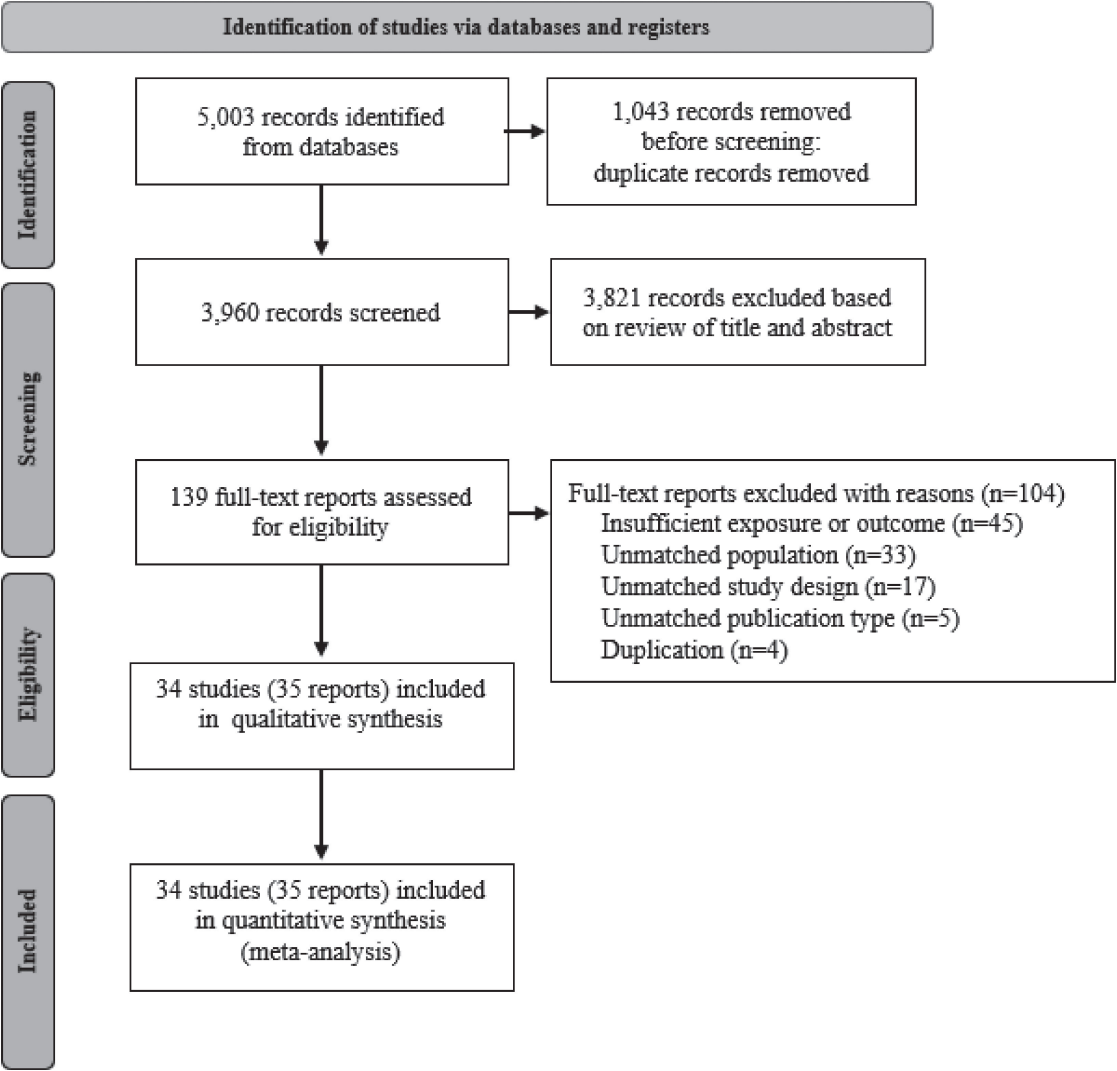
Flow diagram of study selection process.

A total of 5,003 records were identified through database searches. After removing 1,043 duplicates, 3,960 records remained for screening. Of these, 3,821 were excluded based on title and abstract review. The remaining 139 full-text articles were assessed for eligibility, and 104 articles were excluded. Ultimately, 34 studies (35 reports) were included in the systematic review and meta-analysis (Figure 1).^19^^–^^53^ A study population was reported in two separate publications (Wataba 2003^49^ and Wataba 2006^50^), which were combined and treated as a single study.

### Study characteristics

Characteristics of the 34 included studies are presented in Table 1. All studies were conducted in Japan, with publication years ranging from 2002 to 2024. The study designs were predominantly retrospective (*n* = 25),^21^^,^^22^^,^^24^^,^^26^^–^^30^^,^^33^^,^^35^^,^^38^^–^^49^^,^^51^^–^^53^ with some prospective studies (*n* = 5)^23^^,^^25^^,^^34^^,^^36^^,^^37^ and cross-sectional studies (*n* = 1).^20^ Sample sizes ranged from 238 to 419,114 women. Most studies were conducted at a single site (*n* = 24),^19^^,^^20^^,^^23^^–^^27^^,^^30^^–^^33^^,^^35^^,^^36^^,^^38^^–^^42^^,^^45^^–^^47^^,^^49^^,^^52^^,^^53^ while 4 studies were multi-site.^21^^,^^28^^,^^29^^,^^51^ Three studies used a cohort study database,^34^^,^^37^^,^^48^ and two studies used a registry database,^22^^,^^43^ and one utilized a nationwide survey database.^44^

**Table 1.** Characteristics of studies included in systematic review and meta-analyses

	Study, year	Study period, Design	Population		Number of pregnant women (sample size)	Setting	BMI definition of exposure (vs reference normal group BMI)	Number of pregnant women	
	
			Inclusion criteria	Exclusion criteria				Exposed	Not exposed
1	Akamatsu,^19^ 2008	Qualitative research	Pregnant women who delivered vaginally at a participating clinic	Induced, assisted, or aspirated delivery, cesarean section, or previous cesarean section	1,069	single site	BMI <18.5 (BMI 18.5–24.9)	216	808
2	Aoki,^20^ 2010	Cross-sectional study	Pregnant women who delivered in full term and singleton	NR	1,272	single site	BMI <18.5 (BMI 18.5–24.9)	227	916
3	Chihara,^21^ 2010	1998 to 2008, Retrospective study	Healthy nulliparous women with singleton babies in their 20s and 30s	Non-cephalic fetuses and post-term delivery	915	multiple sites	BMI <18.5 (BMI 18.5–24.9)	156	704
4	Enomoto,^22^ 2016	January 1,2013 to December 31, 2013, Retrospective study	Women who delivered at 22 weeks of gestation or later Pregnant women with singleton pregnancies registered in the Japan Society of Obstetrics and Gynecology	Stillbirth, pregnant women with concomitant hypertension or diabetes or other underlying disease, a history of cervical conization, multiple pregnancy, women who delivered a newborn with congenital anomalies, Insufficient data	97,157	registry database	BMI <18.5 (BMI 18.5–24.9)	17,724	69,126
5	Fujimoto,^23^ 2014	June–October 2011, prospective observational study	Pregnant women with singleton pregnancies	Abnormalities or complications affecting fetal development Cases that required emergency cesarean sections and many missing values	238	single site	BMI <18.5 (BMI 18.5–24.9)	58	165

6	Fujiwara,^24^ 2014	January 2001 and December 2012, Retrospective study	Women with a pre-pregnancy BMI of less than 25 kg/m^2^ who delivered live singletons at 22 gestational weeks or more	Those with a history of conization, an uncertain gestational weight gain and those who delivered a newborn with congenital anomalies	8,011	single site	BMI <18.5 (BMI 18.5–24.9)	1,057	6,954
7	Harita,^25^ 2012	November 2006 to April 2008, Prospective cohort study	Woman who delivered full-term singleton babies	Pregnant women who ended in abortion or intrauterine fetal death or miscarriage, Women who did not deliver at the hospital because they were transferred or referred elsewhere, multiple pregnancy, preterm delivery, and insufficient data	1,391	single site	BMI <18.5 (BMI 18.5–24.9)	210	1,086
8	Hyodo,^26^ 2002	1997 to 1999, Retrospective study	Women who gave birth at the target hospital	Women who have miscarried or whose weight is unknown	3,257	single site	BMI ≤18.0 (BMI 18.1–25.0)	358	2,589
9	Imai,^27^ 2004	January 2000 to June 2003, Retrospective study	Single-pregnant women who have delivered after 22 weeks at eligible hospitals	NR	2,424	single site	BMI <18.0 (BMI 18.0–23.9)	278	1,890
10	Ishioka,^28^ 2022	January 2014 to December 2016, Retrospective study	Women with low-risk deliveries	Women who had a premature birth (under 37 weeks of pregnancy) Women who were taken to hospital during pregnancy due to pregnancy-induced hypertension Women who were taken to hospital during labor as a result of arrested labor, non-assuring fetal status Women with abnormal fetal position Women with eclampsia Women with incomplete data records	2,038	multiple sites	Underweight BMI <18.5 (Slightly underweight 18.5 ≦ BMI < 21, Normal 21 ≦ BMI < 23)	Underweight (*n* = 358), Slightly underweight (*n* = 871)	427
11	Kaimura,^29^ 2017	January 2011 and March 2014, Retrospective study	Pregnant women registered in the data system	Multiple births, stillbirths, missing height data	2,939	multiple sites	BMI <18.5 (BMI 18.5–24.9)	406	2,109
12	Kasuga,^30^ 2019	January 2013 to December 2017, Retrospective study	Full-term pregnancies (delivery at or after 37 gestational weeks) in Japanese women who underwent perinatal care at Kawasaki Municipal Hospital.	Pregnancy loss before 22 weeks of gestation, preterm birth (delivery before 37 gestational weeks), multiple pregnancies, women who did not recall their pre-Pregnancy information, pregnancies associated with a congenital anomaly, gestational diabetes mellitus (GDM), or diabetes mellitus before pregnancy, women with missing data	3,237	single site	BMI <18.5 (BMI 18.5–24.9)	566	2,671
13	Kawahara,^31^ 2009	May 23, 2007–May 23, 2008, Relational exploratory study	Pregnant women who had a vaginal delivery after 37 weeks at the target hospital	NR	700	single site	BMI <18.5 (BMI 18.5–24.9)	138	498
14	Kobayashi,^32^ 2010	April 1, 2006 to March 31, 2008, Relational exploratory study	Pregnant women with a singleton delivery	NR	1,098	single site	NR	135	900
15	Murai,^33^ 2017	January 2011 to December 2011, Retrospective study	Mothers with a pre-pregnancy BMI of less than 23 kg/m^2^. Mothers who delivered singleton infants at full term (≥37 weeks and <42 weeks gestation). Mothers without gestational diabetes or pregnancy-induced hypertension.	Multiple pregnancies, Preterm births (<37 weeks), Miscarriages, Stillbirths, Births at ≥42 weeks gestation, Mothers with gestational diabetes, Mothers with pregnancy-induced hypertension, Pre-pregnancy BMI ≥23.0 kg/m^2^, Missing data on drinking, gestational weight gain, and delivery method	1,336	single site	BMI <18.5 (BMI 18.5–22.9)	347	989
16	Murakami,^35^ 2005	During the year 2001, Retrospective study	Women who delivered live singleton babies between 24 and 42 weeks of gestation	NR	633	single site	BMI <18.5 (BMI 18.5–25.0)	94	489
17	Murata,^34^ 2021	August 1, 2011 to mid-2014 Prospective cohort study	Singleton pregnancies Gestation after 22 weeks Participants from the Japan Environment and Children’s Study	Multiple pregnancies Abortion and stillbirth Missing information	71,799	cohort database	BMI <18.5 (BMI 20.0 to <23.0)	10,935	27,835
18	Nagayama,^36^ 2009	January 2006 to December 2006, Prospective study	Pregnant women who have spontaneously delivered a singleton at 37 weeks gestation or later	NR	454	single site	BMI <18.0 (BMI 18.0–24.0)	54	343
19	Nakanishi,^37^ 2022	January 2011 to March 2014, Prospective cohort study	Pregnant individuals from the Japan Environment and Children’s Study dataset (jecs-ta-20190930). Singleton pregnancies. Pregnancies with available data on pre-pregnancy BMI, gestational age at delivery, and neonatal birth weight	Miscarriages Stillbirths Unknown birth outcomes Multiple pregnancies Pregnancies with chromosomal abnormalities Missing data on pre-pregnancy BMI Missing data on gestational age at delivery Missing data on neonatal birth weight Gestational age of <22 weeks or ≥42 weeks (for SGA analysis).	92,260	cohort database	Severe-moderate underweight: BMI <16.9 kg/m^2^. Mild underweight: BMI 17.0–18.4 kg/m^2^. Low-normal weight: BMI 18.5–19.9 kg/m^2^. High-normal weight (reference group): BMI 20.0–22.9 kg/m^2^.	Severe-moderate underweight BMI <16.9 2,515	35,537
20	Nishii,^38^ 2007	April 2004–December 2005, Retrospective study	Uncomplicated singleton pregnancies of 37–41 weeks delivered at eligible hospitals	NR	1,500	single site	BMI <18.0 (BMI 18.0–23.9)	131	1,176
21	Nobumoto,^39^ 2013	January 2009 to June 2012, Retrospective study	Pregnant women who delivered at the hospital	NR	1,121	single site	BMI <18.5 (BMI 18.5–24.9)	216	771
22	Shiina,^40^ 2004	Retrospective study March 21, 1999-May 13, 2002	Pregnant women with a single fetus who gave birth at an eligible hospital	Pregnant women giving birth at home, pregnant women giving birth prematurely at less than 34 weeks	400	single site	BMI <18.0 (BMI 18.0–24.0)	57	291
23	Suzuki,^41^ 2016	January 2011 to December 2011, Retrospective study	Singleton pregnancies delivered at ≥37 weeks gestation	Multiple pregnancies Preterm births (<37 weeks) Miscarriages Stillbirths Post-term births Pre-pregnancy BMI ≥25 Missing data regarding drinking habits, gestational weight gain, and caesarean section	1,518	single site	BMI <18.5 (BMI ≥18.5–25.0)	363	1,155
24	Takahashi,^42^ 2007	2001–2005, Retrospective study	Pregnant women who delivered at 37 weeks gestation or later in full term	Cases of multiple births, cases of obvious fetal anomalies before delivery, amniotic fluid abnormalities, and elective cesarean sections	593	single site	BMI <18.5 (BMI 18.5–24.9)	97	424
25	Takeda,^43^ 2024	2015–2017, Retrospective study	Pregnant women included in the 2015–2017 Japan Society of Obstetrics and Gynecology Perinatal Database	Multiple births. Stillbirths. Deliveries missing data on key variables (number of fetuses, Maternal age, height, pre-pregnancy BMI, complications During pregnancy, gestational age at delivery, birthweight, or mode of delivery). Deliveries from women aged under 18 or over 45 years. Deliveries from women with diagnosed essential hypertension. Deliveries from women with pre-gestational diabetes mellitus. Deliveries from women with previous cesarean delivery. Deliveries from women taking psychotropic drugs.	419,114	registry database	BMI <18.5 (BMI 18.5–24.9)	74,665	303,426
				Deliveries from women who had deliveries under 28 weeks and After 42 weeks of gestation. 6,573 to 5,91 deliveries from women estimated to have a Gestational weight gain rate outside the range of 0–20 kg at 40 weeks. Deliveries from women with missing data on gestational weight gain					
26	Takimoto,^44^ 2006	2000, Retrospective study	Women who delivered from 37 to 41 weeks of age	NR	9,120	nationwide survey database	BMI <18.5 (BMI 18.5–24.9)	1,569	6,840
27	Tanaka,^45^ 2014	January 2010 to January 2013, Retrospective study	Japanese women who delivered at Osaka-Minami Medical Center between 35 and 42 weeks of gestation	Women with multiple gestation. Women with underlying diseases or complications. Women with preterm birth.	1,883	single site	BMI <18.5 (BMI 18.5–22.9)	377	1,204
28	Tani,^46^ 2007	January 2005 to December 2006, Retrospective study	Singleton pregnancies managed for pregnancy and delivery during the study period Non-pregnancy body weight clearly known	Cases of planned cesarean section (excluded from some analyses)	598	single site	BMI <18.0 (BMI 18.0–24.0)	73	440
29	Ueda,^47^ 2007	January 1, 2000, to September 30, 2004, Retrospective study	Pregnant women who received prenatal care from less than 10 weeks of gestation to delivery, and who delivered a singleton at full term.	Women with internal complications, uterine anomalies, fibroids, or previous cesarean section.	502	single site	BMI <18.0 (BMI 18.0–24.0)	47	390
30	Wagata,^48^ 2020	July 2013 to September 2016, Retrospective cohort study	Pregnant women who participated in the Tohoku Medical Megabank Birth and Three-Generation Cohort Study and provided their Maternal and Child Health	Women with no birthweight data or with birth weight <500 g or >6,000 g women with no data for height or pre-pregnancy body weight those with intrauterine fetal demise lack of data at delivery, and multiple pregnancies.	4,810	cohort database	BMI <18.5 (BMI 18.5–24.9)	647	3,590
31	Wataba,^49^ 2006 (Wataba,^50^ 2003)	1981 to 1999, Retrospective cohort study	Pregnant women who delivered a singleton within the eligibility period	Women with cases of preterm labor, multiple births, malformations, and stillbirths	21,719	single site	BMI <18.0 (BMI 18.0–24.0)	2,359	17,100
32	Watanabe,^51^ 2010	January 1, 2003 - December 31, 2004, Retrospective cross-sectional study	Women with BMI <25.0 kg/m^2^ who gave birth to single term infants (37–42 weeks), Deliveries at clinics and hospitals in the Tokyo metropolitan area	Hypertension, preeclampsia, renal disease, gestational diabetes, overweight (BMI 5.0 kg/m^2^), missing information	3,661	multiple sites	BMI <18.5 (BMI 18.5–24.9)	572	2,708
33	Yazaki,^52^ 2012	2003 to 2010, Retrospective study	First-time mothers who delivered after 36 weeks’ gestation Pregnant women with pre-pregnancy BMI and weight gain data at 36 weeks’ gestation	Twin pregnancies, Preterm delivery <36 weeks of gestation	2,083	single site	BMI <18.0 (18.0–23.9)	265	1,597
34	Yoshiike,^53^ 2014	April 1, 2011 - March 31, 2012, Retrospective study	Pregnant women who gave birth at the target hospital during the study period	NR	661	single site	BMI <18.5 (BMI 18.5–24.9)	132	484

BMI, body mass index; NR, not reported; SGA, small for gestational age.

Note: ‘Number of pregnant women’ refers to the entire sample size of the original study. The sum of ‘Exposed (underweight)’ and ‘Not exposed (normal weight)’ may not equal the total, as participants in other BMI categories (eg, overweight, obese) were excluded from this meta-analysis.

The exposure definition was primarily based on pre-pregnancy BMI, with most studies defining underweight as BMI <18.5 kg/m^2^ (*n* = 24),^19^^–^^25^^,^^28^^–^^31^^,^^33^^–^^35^^,^^37^^,^^39^^,^^41^^–^^45^^,^^48^^,^^51^^,^^53^ in accordance with WHO criteria. Some studies used a lower cutoff of BMI <18.0 kg/m^2^ (*n* = 9).^26^^,^^27^^,^^36^^,^^38^^,^^40^^,^^46^^,^^47^^,^^49^^,^^52^ The reference groups were typically women with normal BMI, defined as either 18.5–24.9 kg/m^2^ or 18.0–24.0 kg/m^2^, depending on the study.

A wide range of perinatal outcomes were reported across studies. The most frequently reported outcomes included birth weight parameters (LBW, SGA), PTB, cesarean section, gestational diabetes mellitus, hypertensive disorders of pregnancy, and Apgar scores.

### Quality assessment

The quality of included studies was assessed using NOS, as shown in Table 2. The quality scores ranged from 3 to 7 stars (out of a maximum of 9), with a mean score of 5.2. Seven studies received high scores of 7 stars, indicating high quality.^22^^,^^29^^,^^30^^,^^33^^,^^37^^,^^43^^,^^48^ The majority of studies (18 studies) received 5 or 6 stars, suggesting moderate quality.^19^^,^^21^^,^^23^^–^^25^^,^^28^^,^^31^^,^^32^^,^^34^^–^^36^^,^^41^^,^^42^^,^^44^^,^^48^^,^^49^^,^^51^^,^^53^ Nine studies received lower scores of 3 or 4 stars, indicating lower quality.^20^^,^^26^^,^^27^^,^^38^^–^^40^^,^^46^^,^^47^^,^^52^

**Table 2.** Quality assessment of the included studies according to the Newcastle Ottawa Scale

Study	Selection				Comparability	Outcome			Total number of stars
	
	Representative-ness of the exposed cohort	Selection of the non-exposed cohort	Ascertainment of exposure	Outcome of interest was not present at start of study		Assessment of outcome	Sufficient follow-up time	Adequacy of follow-up	
Akamatsu,^19^ 2008		★	★		★★	★	★		6
Aoki,^20^ 2010		★			★		★		3
Chihara,^21^ 2010	★	★	★		★★		★		6
Enomoto,^22^ 2016	★	★	★		★★	★	★		7
Fujimoto,^23^ 2014		★			★★	★	★	★	6
Fujiwara,^24^ 2014		★	★		★★	★	★		6
Harita,^25^ 2012	★	★			★	★	★		5
Hyodo,^26^ 2002		★			★	★	★		4
Imai,^27^ 2004		★			★		★		3
Ishioka,^28^ 2022		★			★★	★	★	★	6
Kaimura,^29^ 2017	★	★	★		★	★	★	★	7
Kasuga,^30^ 2019		★	★		★★	★	★	★	7
Kawahara,^31^ 2009		★	★		★★	★	★		6
Kobayashi,^32^ 2010		★	★		★	★	★		5
Murai,^33^ 2017		★	★		★★	★	★	★	7
Murata,^34^ 2021	★	★			★	★	★	★	6
Murakami,^35^ 2005		★	★		★	★	★		5
Nagayama,^36^ 2009		★	★		★★	★	★		6
Nakanishi,^37^ 2022	★	★			★★	★	★	★	7
Nishii,^38^ 2007		★			★		★		3
Nobumoto,^39^ 2013		★			★	★	★		4
Shiina,^40^ 2004		★			★		★		3
Suzuki,^41^ 2016		★			★★	★	★		5
Takahashi,^42^ 2007		★	★		★	★	★		5
Takeda,^43^ 2024	★	★	★		★	★	★	★	7
Takimoto,^44^ 2006	★	★			★	★	★		5
Tanaka,^45^ 2014		★	★		★	★	★	★	6
Tani,^46^ 2007		★			★★		★		4
Ueda,^47^ 2007		★			★		★		3
Wagata,^48^ 2020	★	★	★		★	★	★	★	7
Wataba,^49^ 2006 (Wataba,^50^ 2003)		★	★		★	★	★		5
Watanabe,^51^ 2010		★			★	★	★	★	5
Yazaki,^52^ 2012		★			★		★		3
Yoshiike,^53^ 2014		★	★		★	★	★		5

Note: Evaluated by selection, comparability, and outcomes. A study can be awarded a maximum of one star for each numbered item within the Selection and Outcome categories. A maximum of two stars can be given for Comparability. The maximum total score is 9 stars.

Most studies scored well on the “selection of non-exposed cohort” and “sufficient follow-up time” categories. However, variability was observed in the “representativeness of the exposed cohort,” “ascertainment of exposure,” and “adequacy of follow-up” domains. Many studies had limitations in the comparability domain, with inadequate adjustment for confounding factors.

### Publication bias

Funnel plots for the primary outcomes are shown in eFigure 1, eFigure 2, and eFigure 3. Using Egger’s test, there was no evidence of significant publication bias for the OR (*P* = 0.37) and mean difference in birth weight (*P* = 0.18) for LBW outcome, or the mean difference of weeks for PTB outcome (*P* = 0.18). Egger’s test was not performed for the OR of PTB outcome (6 studies) or SGA (9 studies), as fewer than 10 studies were available for analysis.

### Primary outcomes

**Figure 2.**
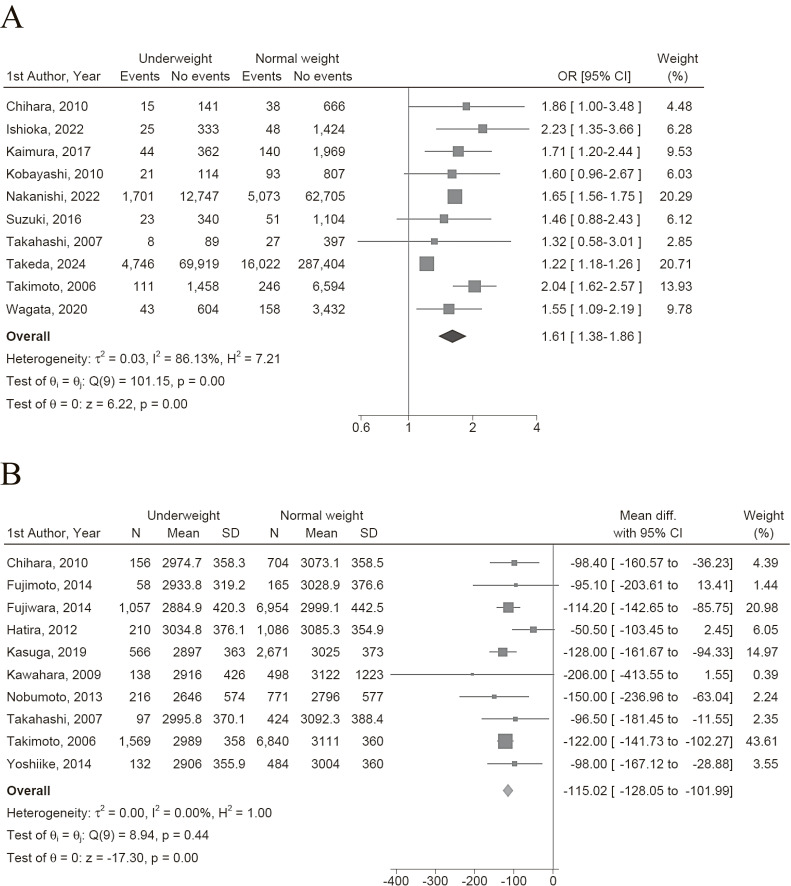
Forest plots showing the association between pre-pregnancy underweight and low birth weight: (**A**) Crude odds ratios for LBW incidence, (**B**) Mean differences in birth weight (grams). CI, confidence interval; OR, odds ratio; SD, standard deviation.

As shown in Figure 2A, maternal pre-pregnancy underweight was significantly associated with an increased risk of LBW compared to normal weight, with a pooled OR of 1.61 (95% CI, 1.38–1.86). This analysis included 10 studies with a total of 481,242 pregnancies.^21^^,^^28^^,^^29^^,^^32^^,^^37^^,^^41^^–^^44^^,^^48^ There was substantial heterogeneity among studies (I^2^ = 86.13%). For comparison, the pooled estimate using a fixed-effects model was OR 1.32 (95% CI, 1.29–1.36).

Mean birth weight was also significantly lower in infants born to underweight mothers compared to normal weight mothers (Figure 2B). The pooled mean difference was −115.02 g (95% CI, −128.05 to −101.99). Similar results were found in a sensitivity analysis including all eligible regardless of BMI definition (OR 1.61; 95% CI, 1.42–1.84; −119.23 g; 95% CI, 131.21 to −107.25 g) (eFigure 4A).

**Figure 3.**
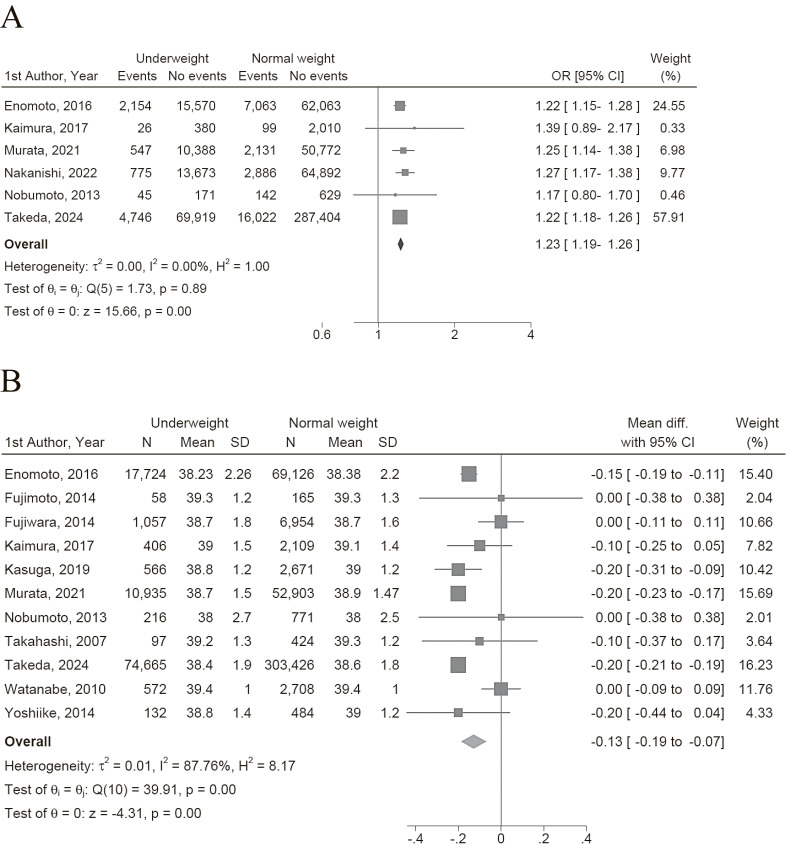
Forest plots showing the association between pre-pregnancy underweight and preterm birth. (**A**) Crude odds ratios for preterm birth incidence comparing underweight and normal weight women. (**B**) Mean differences in gestational age at delivery (weeks). CI, confidence interval; OR, odds ratio; SD, standard deviation.

The association between maternal pre-pregnancy underweight and PTB is presented in Figure 3A. The meta-analysis of six studies showed a significant association between maternal underweight and increased risk of PTB (OR 1.23; 95% CI, 1.19–1.26).^22^^,^^29^^,^^34^^,^^37^^,^^39^^,^^43^ No evidence of heterogeneity was observed among these studies (I^2^ = 0.00%). Mean gestational age at delivery for underweight and normal pre-pregnancy BMI women was reported in 11 studies,^22^^–^^24^^,^^29^^,^^30^^,^^34^^,^^39^^,^^42^^,^^43^^,^^51^^,^^53^ with a pooled mean difference of −0.13 weeks (95% CI, −0.19 to −0.07) (Figure 3B). High heterogeneity observed (I^2^ = 87.76%). For comparison, the pooled estimate using a fixed-effects model was a mean difference of −0.19 weeks (95% CI, −0.20 to −0.18). A sensitivity analysis including all eligible regardless of BMI definition found an OR of 1.23 (95% CI, 1.20–1.26) for PTB, and a mean difference in age at delivery of −0.14 weeks (95% CI, −0.20 to −0.08) (eFigure 5A).

**Figure 4.**
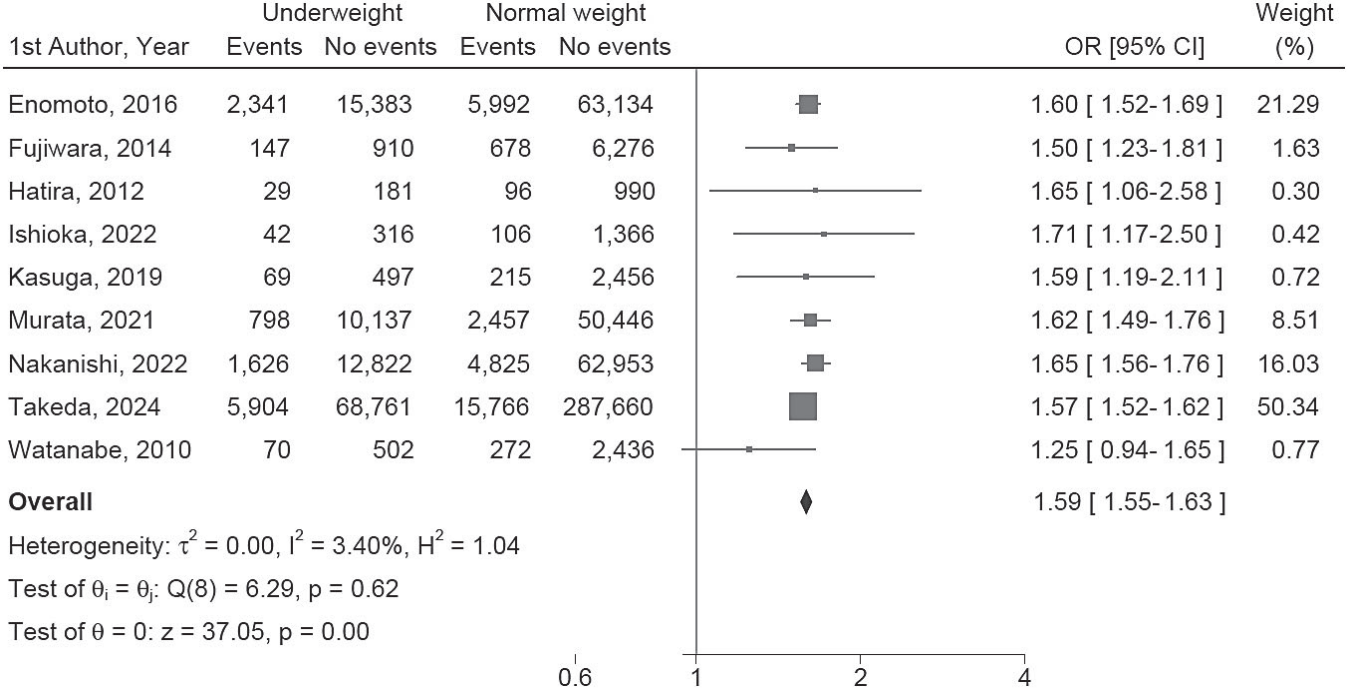
Forest plot of crude odds ratios for small for gestational age comparing pre-pregnancy underweight and normal weight women. CI, confidence interval; OR, odds ratio.

The association between pre-pregnancy underweight and SGA was reported in 9 studies (Figure 4).^22^^,^^24^^,^^25^^,^^28^^,^^30^^,^^34^^,^^37^^,^^43^^,^^51^ Women with pre-pregnancy underweight had a significantly higher risk of delivering an SGA infant compared to women with normal BMI (OR 1.59; 95% CI, 1.55–1.63). Same results were found in a sensitivity analysis (eFigure 6). Statistical heterogeneity was very low (I^2^ = 3.40%). Similar findings were found in the four studies that reported adjusted OR data (OR 1.65; 95% CI, 1.55–1.75, I^2^ = 0.00%).^22^^,^^24^^,^^25^^,^^45^

### Secondary outcomes

Pre-pregnancy underweight was associated with a lower odd of certain maternal complications compared to normal weight, although these findings should be interpreted with caution. The association with gestational diabetes mellitus was not statistically significant (OR 0.76; 95% CI, 0.56–1.02) (eTable 1). Underweight women had a significantly lower crude odds of hypertensive disorders in pregnancy (OR 0.65; 95% CI, 0.50–0.85) based on unadjusted estimates from 11 studies (eTable 2).^22^^,^^24^^,^^29^^,^^36^^,^^41^^–^^43^^,^^45^^,^^47^^,^^48^^,^^53^ Substantial heterogeneity was observed across studies (I^2^ = 88.97%). Cesarean sections were also less common in underweight women (OR 0.80; 95% CI, 0.78–0.82) with no heterogeneity across 13 studies (eTable 3).^21^^,^^22^^,^^26^^,^^27^^,^^29^^,^^34^^,^^36^^,^^40^^,^^43^^,^^45^^,^^47^^,^^52^^,^^53^ A modest reduction in postpartum hemorrhage was noted (OR 0.92; 95% CI, 0.88–0.96), although the pooled mean difference in estimated blood loss was not statistically significant (mean difference −9.43 mL; 95% CI, −29.51 to 10.65) (eTable 4). Underweight women gained slightly more weight than normal weight women during pregnancy (mean difference 0.31; 95% CI, 0.16–0.46 kg) (eTable 5).

For secondary infant outcomes, pre-pregnancy underweight was significantly associated with reduced odds of macrosomia (OR 0.39; 95% CI, 0.33–0.45) (eTable 6) and LGA, both based on five studies with low heterogeneity (eTable 7).^22^^,^^23^^,^^28^^,^^43^^,^^45^ No significant differences were observed in Apgar scores at 1 or 5 minutes (eTable 8) or in the rates of congenital disorders between groups (eTable 9). Evidence on infant hospitalization was limited and inconsistent, with only one study reporting this outcome.^26^

### Subgroup analysis

To explore the substantial heterogeneity observed in the LBW and PTB analysis, we performed subgroup analysis based on the study setting. The pooled estimates were stratified into single-site studies^32^^,^^41^^,^^42^ (OR 1.49; 95% CI, 1.07–2.08), multi-site studies^21^^,^^28^^,^^29^ (OR 1.87; 95% CI, 1.44–2.43), and database studies^37^^,^^43^^,^^44^^,^^48^ (OR 1.56; 95% CI, 1.25–1.95). The test for group differences was not statistically significant for the LBW analyses (*P* = 0.49) (eFigure 7). For the PTB (mean difference) analysis, the test for subgroup differences was statistically significant (*P* = 0.00) (eFigure 8). Among subgroups, database studies^22^^,^^34^^,^^43^ showed a larger effect (mean difference, −0.19) compared to the multi-site^29^^,^^51^ (mean difference, −0.03) and single-site^23^^,^^24^^,^^30^^,^^39^^,^^42^^,^^53^ (mean difference, −0.10) subgroups. Heterogeneity was also higher in the database subgroup than in the other two groups.

## DISCUSSION

This systematic review and meta-analysis represent the first systematic synthesis of evidence examining the association between pre-pregnancy underweight and perinatal outcomes in Japanese women. Across 34 studies, maternal pre-pregnancy underweight was associated with increased risks of adverse infant outcomes, including LBW, PTB, and SGA, along with a reduction in mean birth weight of 115 grams. While crude estimates suggested slightly lower odds of certain maternal complications, such as hypertensive disorders and cesarean section, these findings should be interpreted with caution due to potential confounding and limited adjustment in the included studies.

The clinical significance of our findings is particularly relevant when considering both the magnitude of observed effects and the unique epidemiological context of Japan. Our meta-analysis demonstrated statistically significant increased risks for all primary adverse infant outcomes: LBW (OR 1.61; 95% CI, 1.38–1.86), PTB (OR 1.23; 95% CI, 1.19–1.26), and SGA (OR 1.59; 95% CI, 1.55–1.63). These effect sizes are remarkably consistent with the results of international meta-analyses. For example, Han et al. reported similar risks for LBW (RR 1.64; 95% CI, 1.38–1.94) and PTB (RR 1.29; 95% CI, 1.15–1.46),^3^ while Liu et al’s study in Chinese populations found comparable odds for LBW (OR 1.61; 95% CI, 1.33–1.93) and SGA (OR 1.75; 95% CI, 1.51–2.02).^54^ This consistency is particularly striking given that approximately 20% of reproductive-aged Japanese women are classified as underweight. Notably, the elevated risks of adverse perinatal outcomes remain evident in Japan despite this high background prevalence, highlighting that the harmful impact of maternal underweight is not attenuated in populations where underweight is more common.^55^

Our findings related to secondary maternal outcomes, namely HDP and cesarean section, align with findings from a previous study by Li et al.^56^ A small reduction in postpartum hemorrhage was also observed, which may be partly explained by the lower cesarean section rates among the underweight. The reduced odds of HDP may reflect the absence of obesity-related mechanisms, such as elevated systemic inflammation and insulin resistance.^57^^,^^58^ However, these findings should be interpreted with caution, as confounding factors, such as maternal age, are not accounted for. Moreover, while some maternal complications may be less common in underweight women, the increased risks of fetal growth restriction and its associated long-term consequences^59^^,^^60^ underscore the need for balanced maternal nutrition.

In addition to maternal outcomes, several secondary outcomes were examined. Underweight women gained slightly more weight during pregnancy than normal-weight women, although the differences were modest. This may reflect previous reports and clinical guidance recommending greater gestational weight gain for underweight women to support fetal growth.^10^ Reduced odds of macrosomia and LGA were observed, likely reflecting smaller fetal size associated with maternal undernutrition. However, no significant differences were seen in Apgar scores, infant hospitalization, or congenital anomalies. Only a few studies reported on these outcomes, and their findings were inconsistent, highlighting the need for further research to clarify the potential effects of maternal underweight.

Our analysis revealed substantial heterogeneity for some outcomes, particularly LBW and PTB, while others, including SGA, showed minimal heterogeneity. These heterogeneities likely reflect differences in the study population and methodological approaches across the included studies. The wide temporal span of included studies may also contribute to heterogeneity, given changes over time in prenatal care practices, nutritional awareness, dietary patterns, healthcare systems, and socioeconomic conditions. Nonetheless, the consistency of findings across different BMI classification systems (WHO criteria, BMI <18.5 kg/m^2^ versus other criteria, BMI <18.0 kg/m^2^) in our sensitivity analyses strengthens confidence in our results.

Improving awareness of the perinatal risks associated with pre-pregnancy underweight is critical, given its persistently high prevalence in Japan. Recent studies have shown that the proportion of underweight women has remained stable over time, with a notable concentration in younger age groups.^7^ This secular trend has paralleled an increase in SGA births in Japan.^61^ A contributing factor may be the limited awareness among women regarding the potential consequences of being underweight prior to conception. In a recent nationwide survey, many women of reproductive age were unaware that pre-pregnancy underweight could increase the risk of adverse perinatal outcomes, including SGA.^62^ This highlights the need for targeted education and preconception counseling to improve understanding of healthy weight and its role in reproductive outcomes.

Several limitations in this study must be acknowledged. First, all included studies were observational in nature, limiting the ability to draw causal inferences. Many lacked adjustment for important covariates, leaving a potential for residual confounding. A subgroup analysis by age group was not conducted due to the small number of eligible studies reporting these data. Second, this review excluded studies with fewer than 200 participants to ensure sufficient statistical power and improve the stability of pooled estimates. While this may have resulted in the exclusion of some small-scale studies, the inclusion of several large, high-quality studies utilizing the national perinatal database^22^^,^^43^ likely minimized their impact. These large studies also enhance the generalizability of our findings. Third, exposure classification may have varied across studies due to differences in BMI measurement methods (eg, self-reported, medical records, or non-reported) and a lack of specificity in the timing of assessment, as the included studies simply described “pre-pregnancy” without further detail. Lastly, the substantial heterogeneity observed for some outcomes suggests that pooled estimates should be interpreted with caution, and findings from individual studies may be more informative in certain contexts.

In conclusion, this comprehensive meta-analysis demonstrates that pre-pregnancy underweight among Japanese women is associated with increased risks of LBW, SGA, and PTB. While substantial heterogeneity was observed for some outcomes and warrants cautious interpretation, the overall consistency of these associations, both within this review and in comparison with international reports, underscores the clinical relevance of underweight status. These findings are particularly important in Japan, where the prevalence of underweight in the reproductive-age population is highly prevalent. The consistency of these associations with international evidence reinforces the need for public health attention to underweight status, even in populations where it is relatively common. These findings highlight the importance of addressing underweight as part of preconception care to optimize outcomes for both mothers and their infants.

## SUPPLEMENTARY MATERIAL

eTable 1. Pooled crude odds of gestational diabetes mellitus for underweight versus normal weight women

eTable 2. Pooled crude odds of hypertensive disorders in pregnancy for underweight versus normal weight women

eTable 3. Pooled crude odds of cesarean section for underweight versus normal weight women

eTable 4. Pooled crude odds of postpartum hemorrhage incidence for underweight versus normal weight women

eTable 5. The association of weight gain comparing underweight versus normal weight

eTable 6. Pooled crude odds of macrosomia incidence for underweight versus normal weight women

eTable 7. Pooled crude odds of large for gestational age incidence for underweight versus normal weight women

eTable 8. The association of Apgar scores comparing underweight versus normal weight

eTable 9. Pooled crude odds of congenital disorder incidence for underweight versus normal weight women

eFigure 1. Funnel plots showing the association between pre-pregnancy underweight and low birth weight: (**A**) Crude odds ratios for LBW incidence; (**B**) Mean differences in birth weight. CI, confidence interval; LBW, low birth weight.

eFigure 2. Funnel plots showing the association between pre-pregnancy underweight and preterm birth. (**A**) Crude odds ratios for PTB incidence comparing underweight and normal weight women; (**B**) Mean differences in gestational age at delivery between underweight and normal weight women. CI, confidence interval; PTB, pre-term birth.

eFigure 3. Funnel plots of crude odds ratios for small for gestational age comparing pre-pregnancy underweight and normal weight women. CI, confidence interval.

eFigure 4. Forest plots showing the association between pre-pregnancy underweight and low birth weight: (**A**) Crude odds ratios for LBW incidence, (**B**) Mean differences in birth weight. CI, confidence interval; OR, odds ratio; SD, standard deviation.

eFigure 5. Forest plots showing the association between pre-pregnancy underweight and preterm birth. (**A**) Crude odds ratios for PTB incidence comparing underweight and normal weight women. (**B**) Mean differences in gestational age at delivery between underweight and normal weight women. CI, confidence interval; OR, odds ratio; SD, standard deviation.

eFigure 6. Forest plot of crude odds ratios for small for gestational age comparing pre-pregnancy underweight and normal weight women. CI, confidence interval; OR, odds ratio.

eFigure 7. Subgroup analysis: forest plot showing the association between pre-pregnancy underweight and low birth weight, crude odds ratios for LBW incidence. CI, confidence interval; LBW, low birth weight; OR, odds ratio.

eFigure 8. Subgroup analysis: forest plot showing the association between pre-pregnancy underweight and preterm birth, Mean differences in gestational age at delivery between underweight and normal weight women. CI, confidence interval; OR, odds ratio; SD, standard deviation.

eMaterial 1. Search terms for MEDLINE

eMaterial 2. Search terms for EMBASE

eMaterial 3. Search terms for CINAHL

eMaterial 4. Search terms for PsycINFO

eMaterial 5. Search terms for Cochrane Library (CENTRAL)

eMaterial 6. Search terms for *Ichushi*
