# Supplementary material for: Impact of Maternal Underweight on Infant and Maternal Outcomes in Japanese Women: A Systematic Review and Meta-analysis
Source: J Epidemiol. 2026 Aug 5;36(8):243–56. doi: 10.2188/jea.JE20250483 (PMC13349791; doi:10.2188/jea.JE20250483)
Supplement: Supplementary file 1 [file je-36-243-s001.pdf]

**eTable 1.** Pooled crude odds of gestational diabetes mellitus for underweight versus normal weight women

| Study | Underweight | | Normal weight | | OR (95% CI) | Weight (%) | $I^2$ (%) |
|---|---|---|---|---|---|---|---|
| | Events | No events | Events | No events | | | |
| Enomoto, 2016 | 472 | 17,252 | 2,665 | 66,461 | 0.68 (0.62–0.75) | 43.4 | |
| Fujiwara, 2014 | 89 | 968 | 570 | 6,384 | 1.03 (0.82–1.30) | 35.7 | |
| Kaimura, 2017 | 1 | 405 | 13 | 2,096 | 0.40 (0.05–3.05) | 2.1 | |
| Takahashi, 2007 | 1 | 96 | 6 | 418 | 0.73 (0.09–6.10) | 1.9 | |
| Tanaka, 2014 | 2 | 375 | 13 | 1,191 | 0.49 (0.11–2.18) | 3.7 | 50.3 |
| Ueda, 2007 | 0 | 47 | 0 | 390 | 8.22 (0.16–419.13) | 0.6 | |
| Wataba, 2003 | 5 | 2,354 | 65 | 17,035 | 0.56 (0.22–1.38) | 8.8 | |
| Yoshiike, 2014 | 2 | 130 | 16 | 468 | 0.45 (0.10–1.98) | 3.8 | |
| Overall | 572 | 22,293 | 3,348 | 98,280 | 0.76 (0.56–1.02) | - | |

CI, confidence interval; OR, odds ratio.

**eTable 2.** Pooled crude odds of hypertensive disorders in pregnancy for underweight versus normal weight women

| Study | Underweight | | Normal weight | | OR (95% CI) | Weight (%) | $I^2$ |
|---|---|---|---|---|---|---|---|
| | Events | No events | Events | No events | | | |
| Enomoto, 2016 | 592 | 17,132 | 3237 | 65,889 | 0.70 (0.64–0.77) | 18.9 | 89.0 |
| Fujiwara, 2014 | 19 | 1,038 | 382 | 6,572 | 0.32 (0.20–0.50) | 12.0 | |
| Kaimura, 2017 | 10 | 396 | 89 | 2,020 | 0.57 (0.30–1.11) | 8.7 | |
| Nagayama, 2009 | 3 | 51 | 3 | 340 | 6.67 (1.31–33.93) | 2.3 | |
| Suzuki, 2016 | 6 | 357 | 54 | 1,101 | 0.34 (0.15–0.80) | 6.4 | |
| Takahashi, 2007 | 6 | 91 | 37 | 387 | 0.69 (0.28–1.68) | 6.0 | |
| Takeda, 2024 | 1,718 | 72,947 | 9,678 | 293,748 | 0.72 (0.68–0.75) | 19.1 | |
| Tanaka, 2014 | 6 | 371 | 27 | 1,177 | 0.71 (0.29–1.72) | 6.0 | |
| Ueda, 2007 | 1 | 46 | 8 | 382 | 1.04 (0.13–8.49) | 1.5 | |
| Wagata, 2020 | 57 | 590 | 346 | 3,244 | 0.91 (0.68–1.22) | 15.5 | |
| Yoshiike, 2014 | 3 | 129 | 22 | 462 | 0.49 (0.14–1.66) | 3.7 | |
| Overall | 2,421 | 93,148 | 13,883 | 375,322 | 0.65 (0.50–0.85) | - | |

CI, confidence interval; OR, odds ratio.

**eTable 3.** Pooled crude odds of cesarean section for underweight versus normal weight women

| Study | Underweight | | Normal weight | | OR (95% CI) | Weight (%) | $I^2$ |
|---|---|---|---|---|---|---|---|
| | Events | No events | Events | No events | | | |
| Chihara, 2010 | 7 | 149 | 71 | 633 | 0.42 (0.19–0.93) | 0.1 | 0.0 |
| Enomoto, 2016 | 3,995 | 13,729 | 18,627 | 50,499 | 0.79 (0.76–0.82) | 33.6 | |
| Hyodo, 2002 | 54 | 304 | 394 | 2,195 | 0.99 (0.73–1.35) | 0.5 | |
| Imai, 2004 | 22 | 256 | 177 | 1,713 | 0.83 (0.52–1.32) | 0.2 | |
| Kaimura, 2017 | 60 | 346 | 421 | 1,688 | 0.70 (0.52–0.93) | 0.6 | |
| Murata, 2021 | 1,618 | 9,317 | 9,493 | 43,410 | 0.79 (0.75–0.84) | 15.6 | |
| Nagayama, 2009 | 6 | 48 | 18 | 325 | 2.26 (0.85–5.97) | 0.1[a] | |
| Shiina, 2004 | 8 | 49 | 41 | 242 | 0.96 (0.43–2.18) | 0.1 | |
| Takeda, 2024 | 4,660 | 70,005 | 23,150 | 280,276 | 0.81 (0.78–0.83) | 48.4[a] | |
| Tanaka, 2014 | 16 | 361 | 78 | 1,126 | 0.64 (0.37–1.11) | 0.2[a] | |
| Ueda, 2007 | 1 | 46 | 34 | 356 | 0.23 (0.03–1.70) | 0.0 | |
| Yazaki, 2012 | 43 | 222 | 371 | 1,226 | 0.64 (0.45–0.91) | 0.4 | |
| Yoshiike, 2014 | 24 | 108 | 79 | 405 | 1.14 (0.69–1.89) | 0.2 | |
| Overall | 10,514 | 94,940 | 52,954 | 384,094 | 0.80 (0.78–0.82) | - | |

CI, confidence interval; OR, odds ratio.

[a] Emergency Cesarean section

**eTable 4.** Pooled crude odds of postpartum hemorrhage incidence for underweight versus normal weight women

**A)** Comparing crude data on the risk of postpartum hemorrhage incidence

| Study | Underweight | | Normal weight | | OR (95% CI) | Weight (%) | $I^2$ |
|---|---|---|---|---|---|---|---|
| | Events | No events | Events | No events | | | |
| Enomoto, 2016 | 3,468 | 14,256 | 14,476 | 54,650 | 0.92 (0.88–0.96) | 99.0 | |
| Tanaka, 2014 | 30 | 347 | 110 | 1,094 | 0.86 (0.56–1.31) | 1.0 | 0.0 |
| Overall | 3,498 | 14,603 | 14,586 | 55,744 | 0.92 (0.88–0.96) | - | |

**B)** Mean differences in postpartum hemorrhage incidence

| Study | Underweight | | | Normal weight | | | Mean difference | 95% CI | | Weight (%) | $I^2$ |
|---|---|---|---|---|---|---|---|---|---|---|---|
| | Number | Mean | SD | Number | Mean | SD | | Lower | Upper | | |
| Imai, 2004 | 278 | 190.62 | 184.44 | 1,890 | 192.61 | 186.17 | -1.99 | -25.40 | 21.42 | 73.6 | |
| Nagayama, 2009 | 54 | 399.80 | 302.20 | 343 | 431.60 | 329.10 | -31.80 | -125.24 | 61.64 | 4.6 | |
| Shiina, 2004 | 57 | 465.00 | 285.00 | 283 | 491.00 | 312.00 | -26.00 | -113.55 | 61.55 | 5.3 | 0.0 |
| Takahashi, 2007 | 97 | 449.00 | 386.80 | 424 | 464.50 | 403.10 | -15.50 | -103.77 | 72.77 | 5.2 | |
| Yoshiike, 2014 | 132 | 431.00 | 315.50 | 484 | 469.00 | 307.30 | -38.00 | -97.48 | 21.48 | 11.4 | |
| | 618 | | | 3,424 | | | -9.43 | -29.51 | 10.65 | - | |

CI, confidence interval; OR, odds ratio; SD, standard deviation.

**eTable 5.** The association of weight gain comparing underweight versus normal weight

| Study | Underweight | | | Normal weight | | | Mean difference | 95% CI | | Weight (%) | $I^2$ |
|---|---|---|---|---|---|---|---|---|---|---|---|
| | Number | Mean | SD | Number | Mean | SD | | Lower | Upper | | |
| Fujimoto, 2014 | 58 | 10.50 | 3.30 | 165 | 9.90 | 3.60 | 0.60 | -0.46 | 1.66 | 1.8 | |
| Fujiwara, 2014 | 1,057 | 10.40 | 3.80 | 6,954 | 10.10 | 3.70 | 0.30 | 0.06 | 0.54 | 13.4 | |
| Hyodo, 2002 | 358 | 10.60 | 3.60 | 2,589 | 10.20 | 4.20 | 0.40 | -0.06 | 0.86 | 7.1 | |
| Imai, 2004 | 278 | 10.50 | 3.10 | 1,890 | 10.60 | 3.50 | -0.10 | -0.54 | 0.34 | 7.5 | |
| Ishioka, 2022 | 358 | 11.00 | 3.30 | 1,475 | 11.00 | 3.36 | 0.00 | -0.39 | 0.39 | 8.7 | |
| Kaimura, 2017 | 406 | 11.40 | 3.40 | 2,109 | 10.90 | 3.60 | 0.50 | 0.12 | 0.88 | 8.9 | |
| Nagayama, 2009 | 54 | 11.10 | 3.15 | 343 | 10.50 | 3.45 | 0.60 | -0.38 | 1.58 | 2.1 | |
| Suzuki, 2016 | 363 | 11.03 | 3.30 | 1,155 | 10.25 | 3.49 | 0.78 | 0.37 | 1.19 | 8.2 | 51.7 |
| Takahashi, 2007 | 97 | 10.56 | 3.95 | 424 | 10.20 | 3.85 | 0.36 | -0.49 | 1.21 | 2.7 | |
| Takeda, 2024 | 74,665 | 10.10 | 3.30 | 303,426 | 9.90 | 3.60 | 0.20 | 0.17 | 0.23 | 20.4 | |
| Tani, 2007 | 73 | 10.60 | 3.40 | 440 | 11.30 | 4.50 | -0.70 | -1.78 | 0.38 | 1.8 | |
| Ueda, 2007 | 47 | 10.90 | 2.80 | 390 | 9.80 | 3.50 | 1.10 | 0.06 | 2.14 | 1.9 | |
| Watanabe, 2010 | 572 | 10.50 | 3.30 | 1,591 | 10.00 | 3.20 | 0.50 | 0.19 | 0.81 | 11.0 | |
| Yoshiike, 2014 | 132 | 10.10 | 3.20 | 484 | 10.10 | 3.20 | 0.00 | -0.62 | 0.62 | 4.6 | |
| | 78,518 | | | 323,435 | | | 0.31 | 0.16 | 0.46 | - | |

CI, confidence interval; SD, standard deviation.

**eTable 6.** Pooled crude odds of macrosomia incidence for underweight versus normal weight women

| Study | Underweight | | Normal weight | | OR (95% CI) | Weight (%) | $I^2$ |
|---|---|---|---|---|---|---|---|
| | Events | No events | Events | No events | | | |
| Enomoto, 2016 | 47 | 17,677 | 474 | 68,652 | 0.39 (0.29–0.52) | 24.6 | 0.0 |
| Imai, 2004 | 0 | 278 | 14 | 1,876 | 0.23 (0.01–3.91) | 0.3 | |
| Kaimura, 2017 | 4 | 402 | 19 | 2,090 | 1.10 (0.37–3.23) | 1.9 | |
| Takeda, 2024 | 134 | 74,531 | 1,419 | 302,007 | 0.38 (0.32–0.46) | 70.5 | |
| Takimoto, 2006 | 5 | 1,564 | 75 | 6,765 | 0.29 (0.12–0.71) | 2.7 | |
| Overall | 190 | 94,452 | 2,001 | 381,390 | 0.39 (0.33–0.45) | - | |

CI, confidence interval; OR, odds ratio.

**eTable 7.** Pooled crude odds of large for gestational age incidence for underweight versus normal weight women

| Study | Underweight | | Normal weight | | OR (95% CI) | Weight (%) | $I^2$ |
|---|---|---|---|---|---|---|---|
| | Events | No events | Events | No events | | | |
| Enomoto, 2016 | 967 | 16,757 | 6,944 | 62,182 | 0.52 (0.48–0.55) | 32.5 | |
| Fujimoto, 2014 | 5 | 53 | 8 | 157 | 1.85 (0.58–5.91) | 0.2 | |
| Ishioka, 2022 | 23 | 335 | 156 | 1,319 | 0.58 (0.37–0.91) | 1.0 | 10.0 |
| Takeda, 2024 | 2,691 | 71,974 | 19,498 | 283,928 | 0.54 (0.52–0.57) | 65.6 | |
| Tanaka, 2014 | 19 | 358 | 103 | 1,101 | 0.57 (0.34–0.94) | 0.8 | |

CI, confidence interval; OR, odds ratio.

**eTable 8.** The association of Apgar scores comparing underweight versus normal weight

**A**) Comparing crude data on the Apgar scores

| Study | Underweight | | Normal weight | | OR (95% CI) | Weight (%) | $I^2$ |
|---|---|---|---|---|---|---|---|
| | Events | No events | Events | No events | | | |
| Takahashi, 2007 | 11 | 86 | 37 | 387 | 1.34 (0.66–2.73) | 47.2[a] | |
| Tanaka, 2014 | 2 | 375 | 7 | 1,197 | 0.91 (0.19–4.41) | 9.6[a] | |
| Watanabe, 2010 | 8 | 564 | 26 | 1,565 | 0.85 (0.38–1.90) | 37.6[b] | 0.0 |
| Watanabe, 2010 | 1 | 571 | 10 | 1,581 | 0.28 (0.04–2.17) | 5.7[a] | |
| Overall | 22 | 1,596 | 80 | 4,730 | 1.00 (0.61–1.63) | - | |

**B**) Mean differences in Apgar scores in 1 minute.

| Study | Underweight | | | Normal weight | | | Mean difference | 95% CI | | Weight (%) | $I^2$ |
|---|---|---|---|---|---|---|---|---|---|---|---|
| | Number | Mean | SD | Number | Mean | SD | | Lower | Upper | | |
| Hyodo, 2002 | 358 | 7.60 | 1.90 | 2,589 | 7.70 | 1.70 | -0.10 | -0.29 | 0.09 | 22.6 | |
| Imai, 2004 | 278 | 9.10 | 1.00 | 1,890 | 9.00 | 1.10 | 0.10 | -0.04 | 0.24 | 33.0 | |
| Tani, 2007 | 73 | 8.30 | 0.90 | 440 | 8.30 | 1.00 | 0.00 | -0.24 | 0.24 | 16.0 | 38.8 |
| Yoshiike, 2014 | 132 | 8.10 | 0.90 | 484 | 8.20 | 0.80 | -0.10 | -0.26 | 0.06 | 28.4 | |
| | 841 | | | 5,403 | | | -0.02 | -0.13 | 0.09 | - | |

**C**) Mean differences in Apgar scores in 5 minutes.

| Study | Underweight | | | Normal weight | | | Mean difference | 95% CI | | Weight (%) | $I^2$ |
|---|---|---|---|---|---|---|---|---|---|---|---|
| | Number | Mean | SD | Number | Mean | SD | | Lower | Upper | | |
| Hyodo, 2002 | 358 | 8.50 | 1.50 | 2,589 | 8.60 | 1.30 | -0.10 | -0.25 | 0.05 | 29.3 | |
| Shiina, 2004 | 57 | 8.15 | 1.13 | 283 | 8.33 | 0.81 | -0.18 | -0.43 | 0.07 | 10.4 | |
| Tani, 2007 | 73 | 8.70 | 0.60 | 440 | 8.80 | 0.90 | -0.10 | -0.31 | 0.11 | 14.0 | 2.3 |
| Yoshiike, 2014 | 132 | 9.20 | 0.60 | 484 | 9.20 | 0.60 | 0.00 | -0.12 | 0.12 | 46.3 | |
| | 620 | | | 3,796 | | | -0.06 | -0.14 | 0.02 | - | |

CI, confidence interval; OR, odds ratio; SD, standard deviation.

[a] Apgar score of 7 or less at 5 minutes.

[b] Apgar score of 7 or less at 1 minute.

**eTable 9.** Pooled crude odds of congenital disorder incidence for underweight versus normal weight women

| Study | Underweight | | Normal weight | | OR (95% CI) | Weight (%) | $I^2$ |
|---|---|---|---|---|---|---|---|
| | Events | No events | Events | No events | | | |
| Hyodo, 2002 | 16 | 342 | 89 | 2,500 | 1.31 (0.76–2.26) | 86.9 | |
| Takahashi, 2007 | 3 | 94 | 6 | 418 | 2.22 (0.55–9.05) | 13.1 | 0.0 |
| Overall | 19 | 436 | 95 | 2,918 | 1.41 (0.85–2.34) | - | |

CI, confidence interval; OR, odds ratio.

A

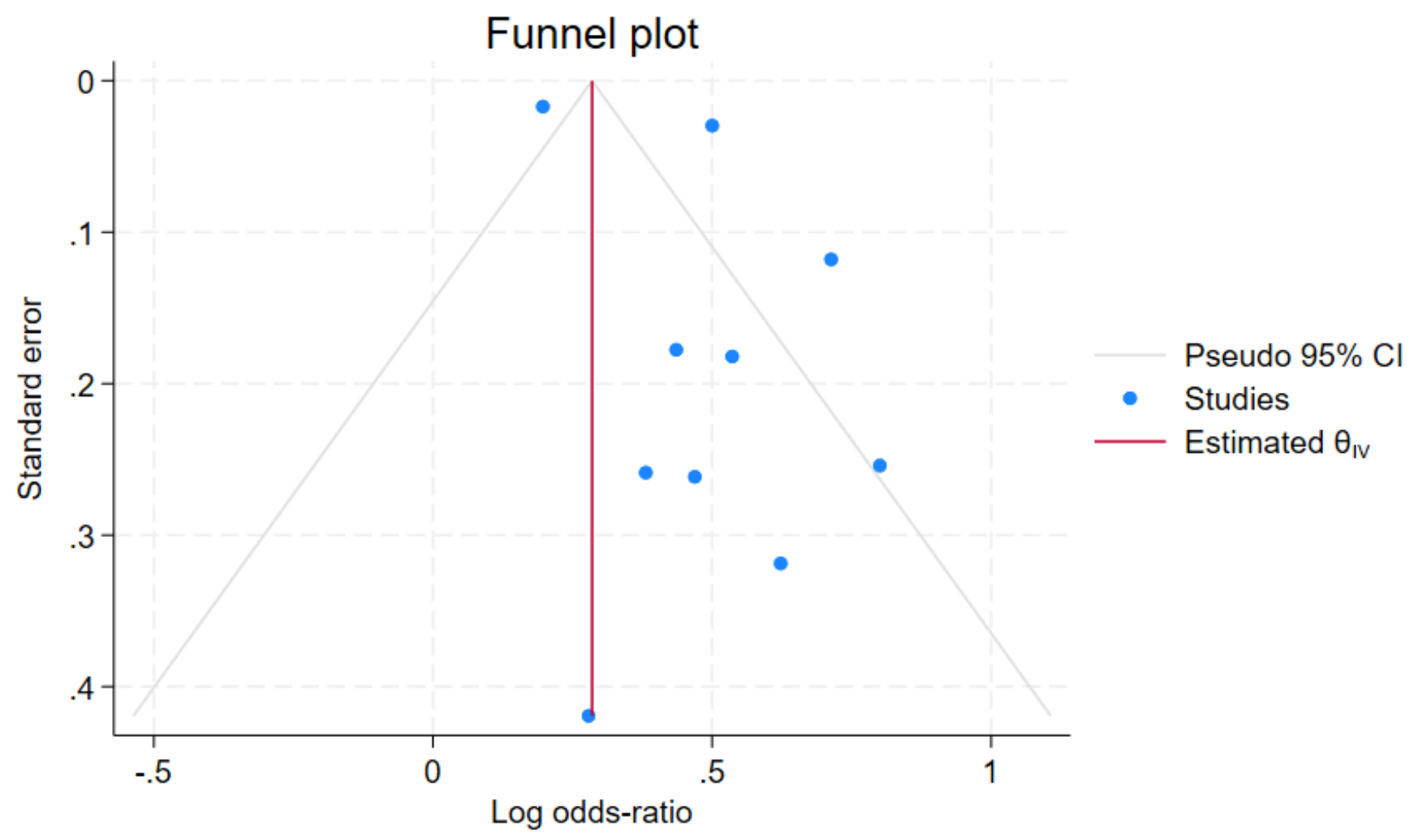


10 studies
Egger’s test: p-value 0.37

B

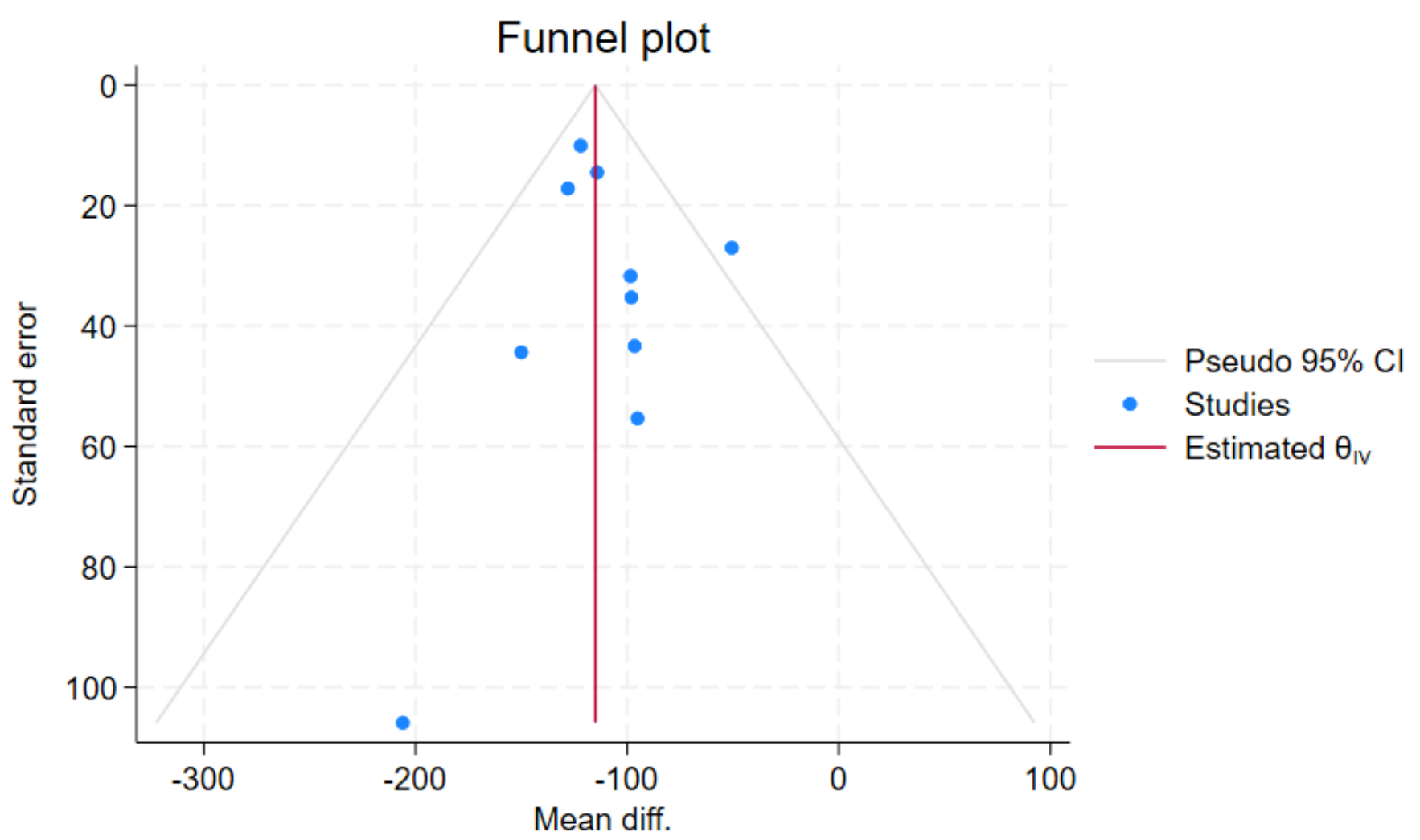


10 studies
Egger’s test: p-value 0.18

**eFigure 1.** Funnel plots showing the association between pre-pregnancy underweight and low birth weight: (**A**) Crude odds ratios for LBW incidence; (**B**) Mean differences in birth weight. CI, confidence interval; LBW, low birth weight.

A

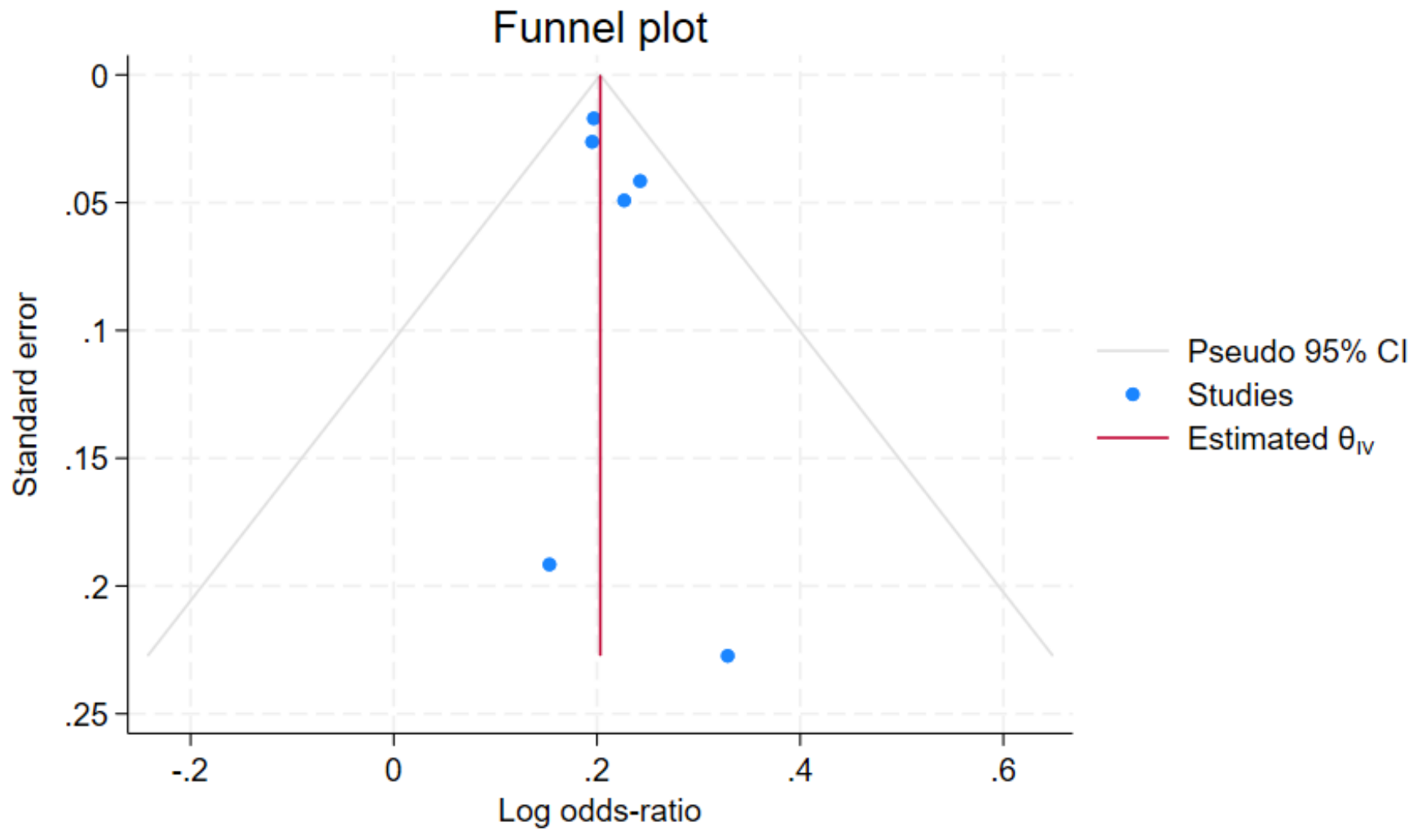


6 studies

B

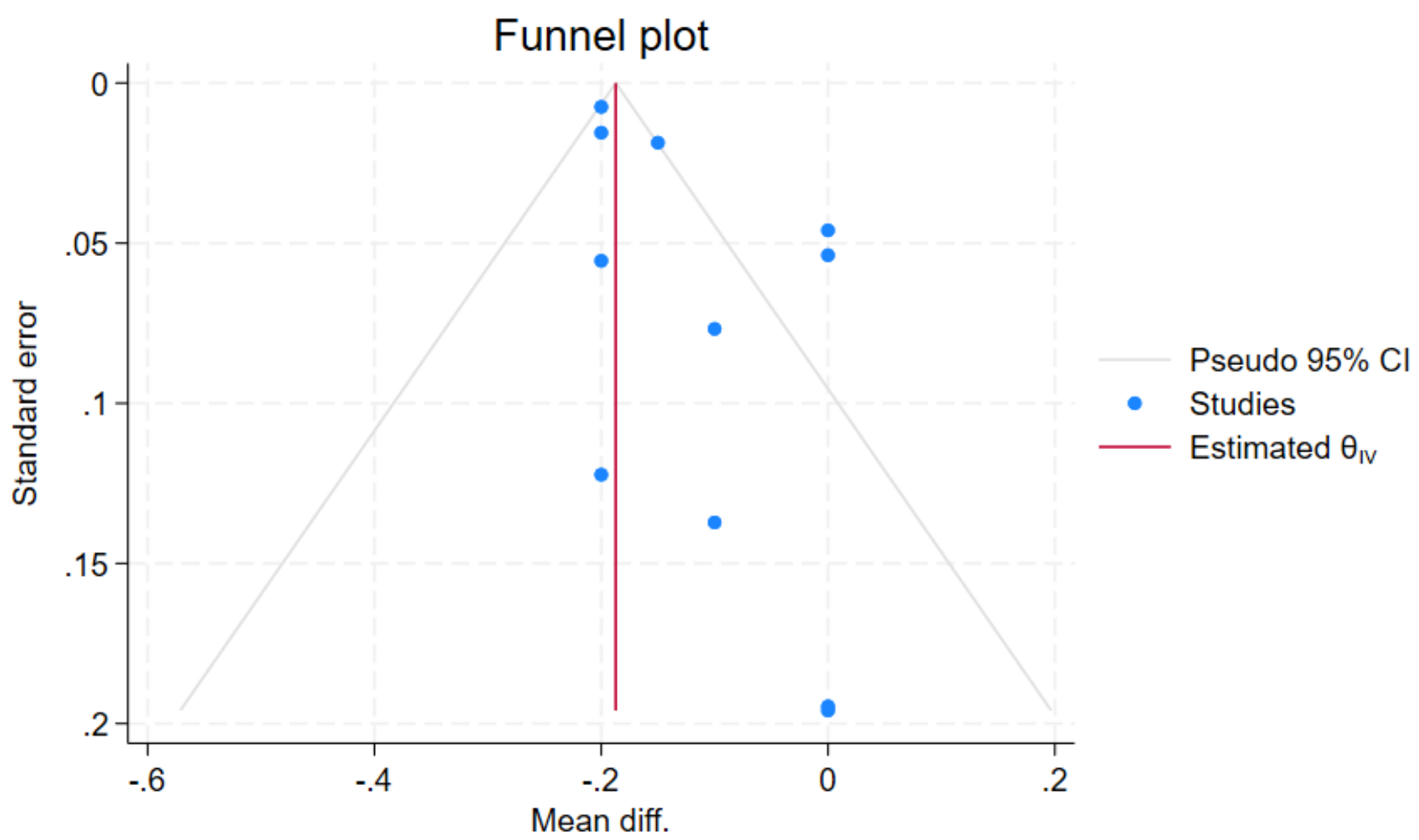


11 studies
Egger's test: p-value 0.18

**eFigure 2.** Funnel plots showing the association between pre-pregnancy underweight and preterm birth. (**A**) Crude odds ratios for PTB incidence comparing underweight and normal weight women; (**B**) Mean differences in gestational age at delivery between underweight and normal weight women. CI, confidence interval; PTB, pre-term birth.

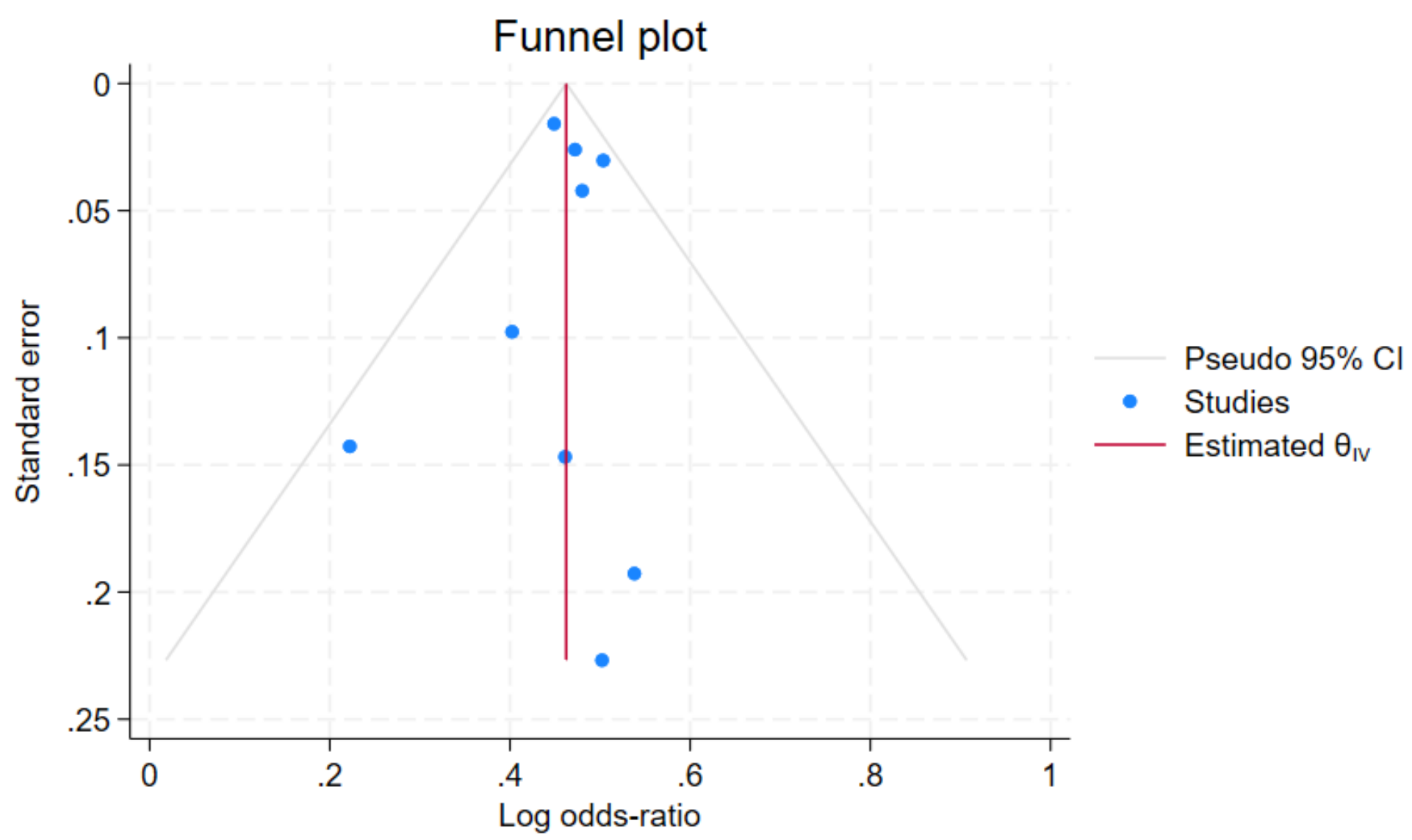


9 studies

**eFigure 3.** Funnel plots of crude odds ratios for small for gestational age comparing pre-pregnancy underweight and normal weight women. CI, confidence interval.

A

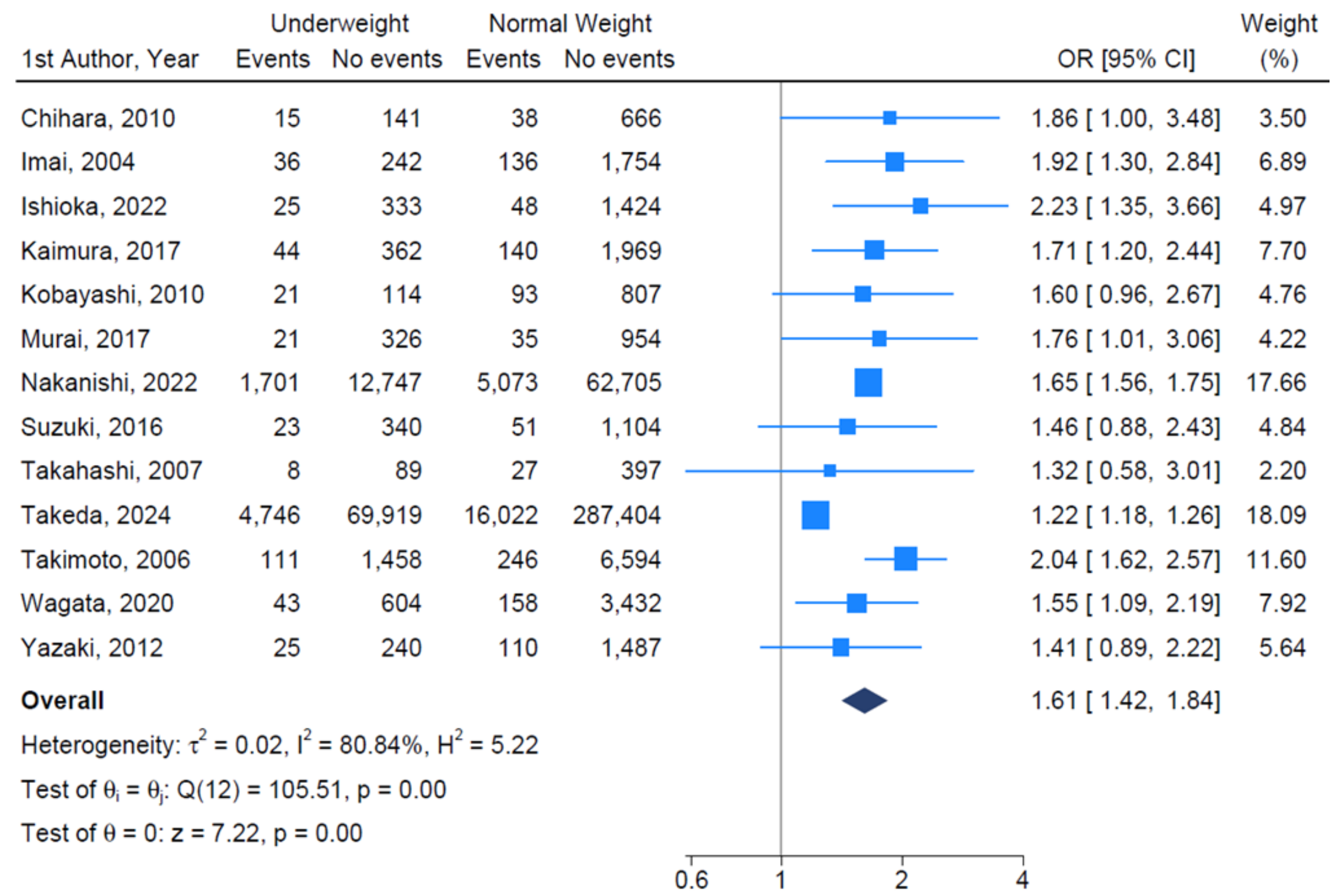


B

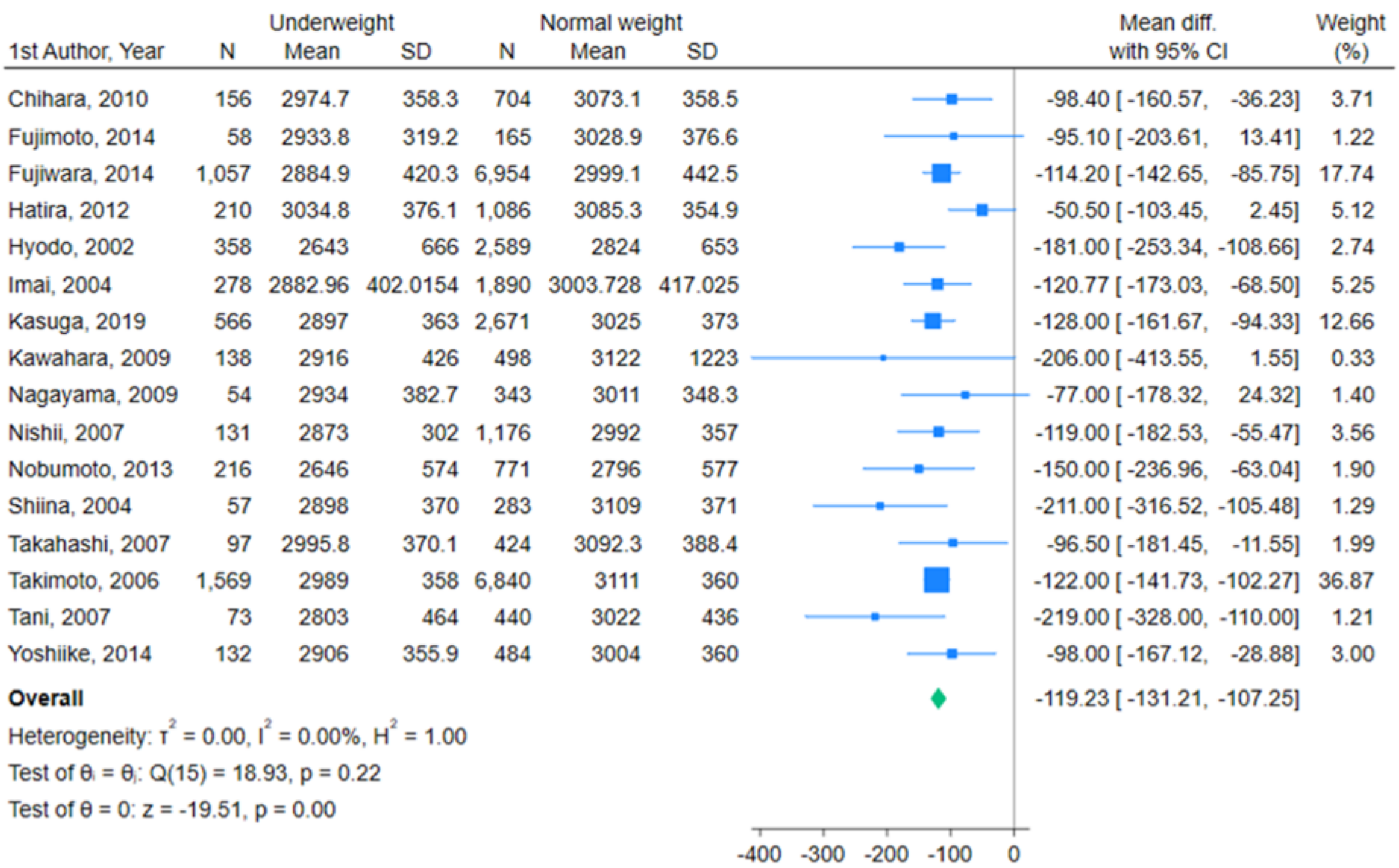


**eFigure 4.** Forest plots showing the association between pre-pregnancy underweight and low birth weight: (**A**) Crude odds ratios for LBW incidence, (**B**) Mean differences in birth weight. CI, confidence interval; OR, odds ratio; SD, standard deviation.

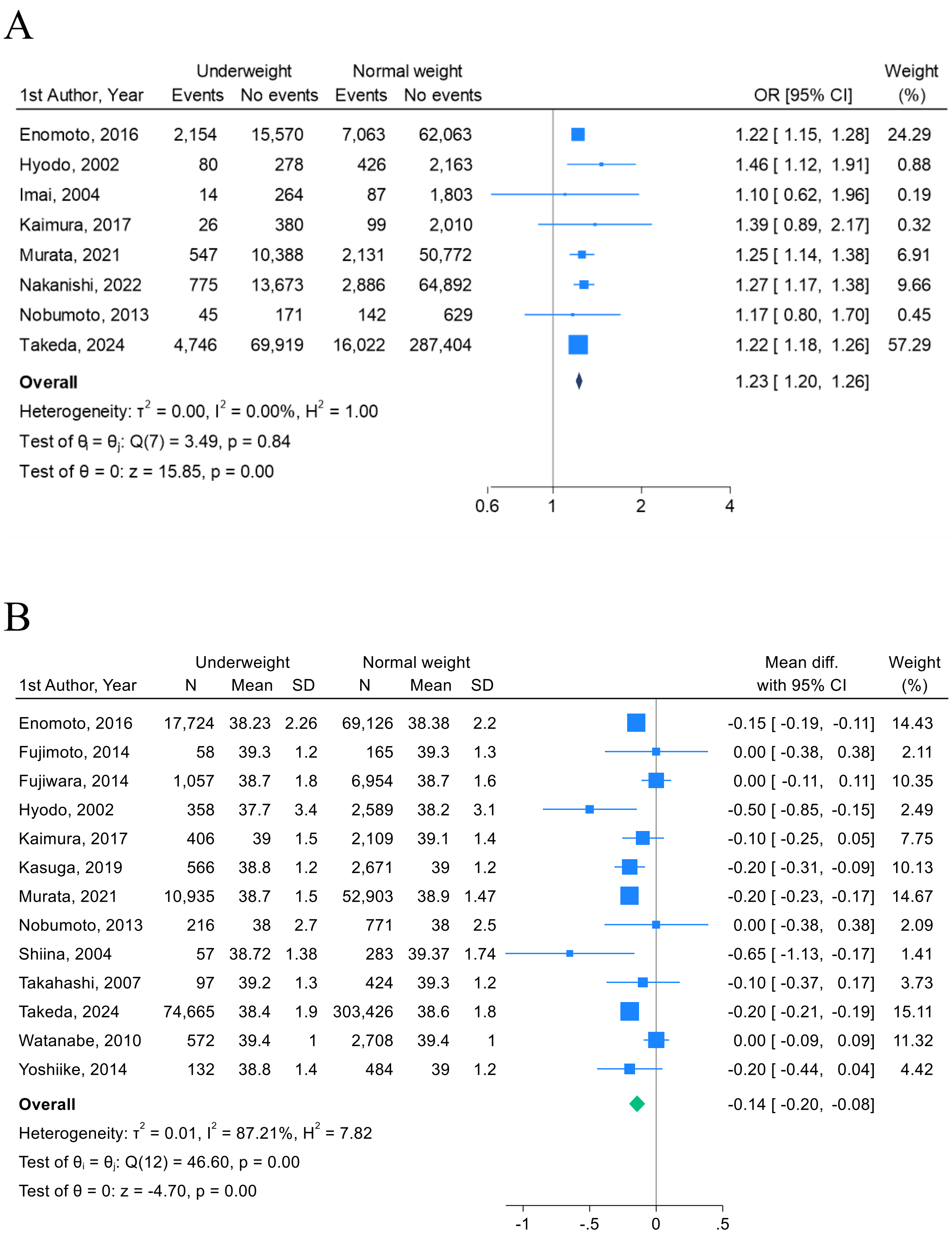


**eFigure 5.** Forest plots showing the association between pre-pregnancy underweight and preterm birth. (**A**) Crude odds ratios for PTB incidence comparing underweight and normal weight women. (**B**) Mean differences in gestational age at delivery between underweight and normal weight women.

CI, confidence interval; OR, odds ratio; SD, standard deviation.

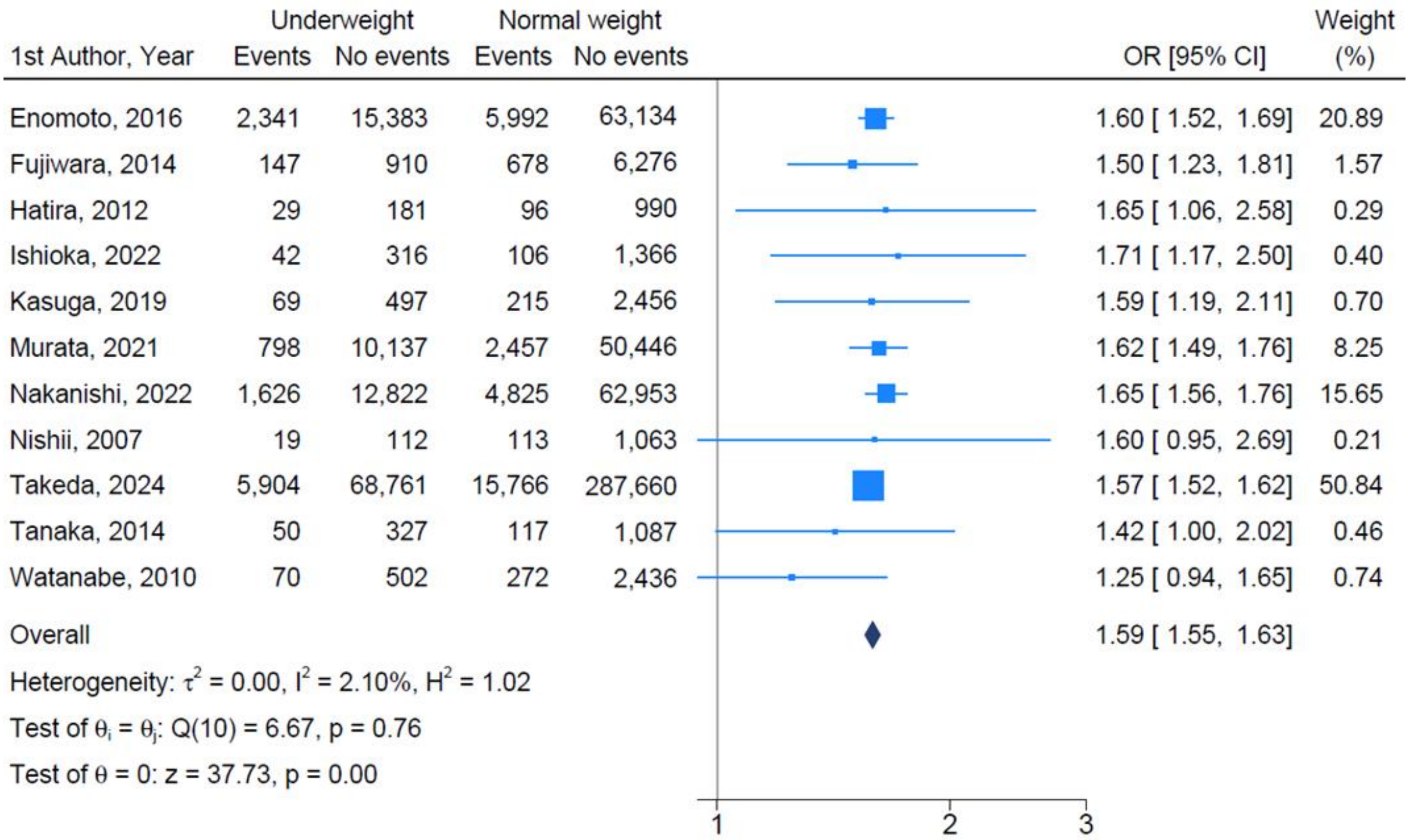


**eFigure 6.** Forest plot of crude odds ratios for small for gestational age comparing pre-pregnancy underweight and normal weight women. CI, confidence interval; OR, odds ratio.

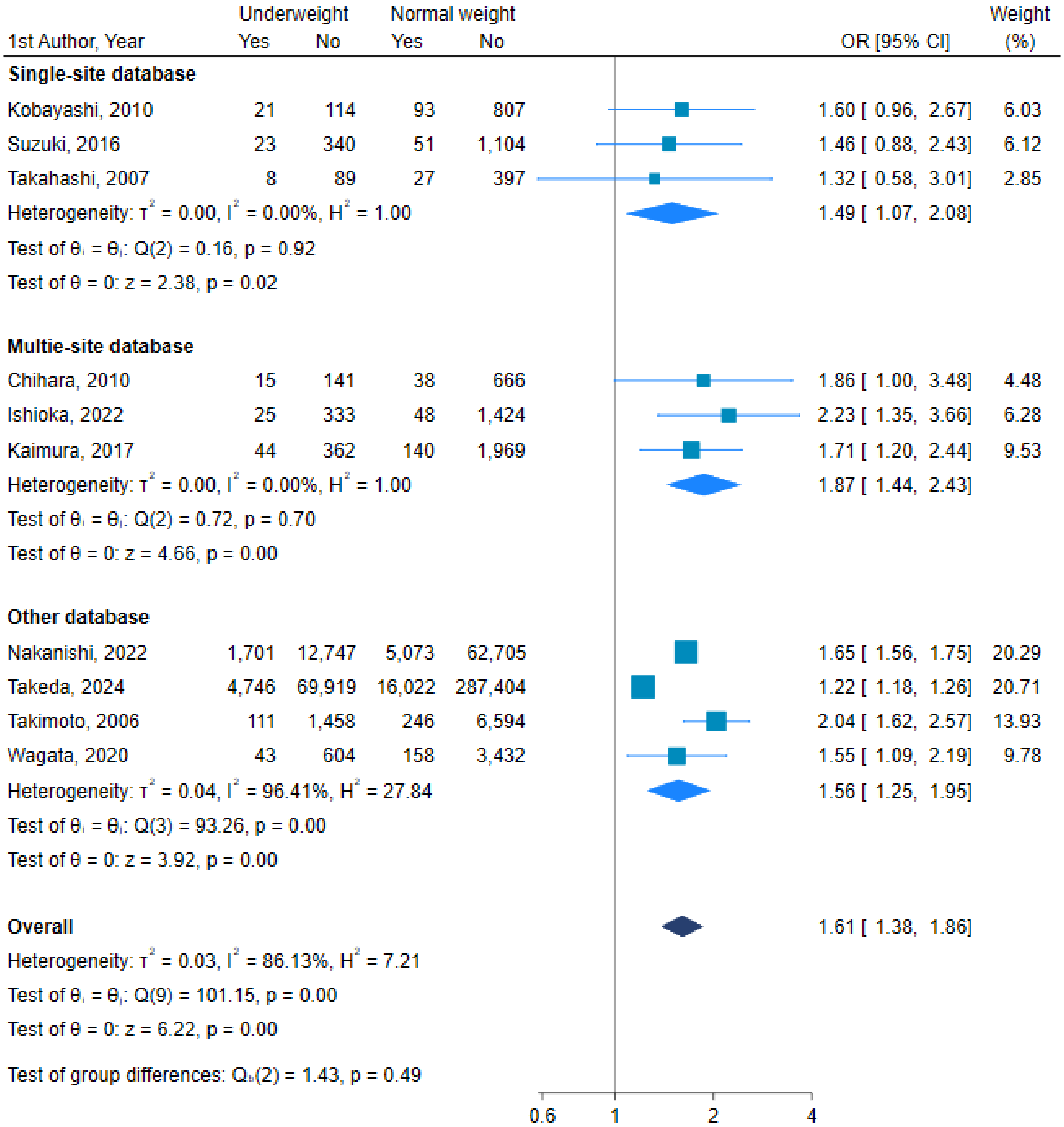


**eFigure 7.** Subgroup analysis: forest plot showing the association between pre-pregnancy underweight and low birth weight, crude odds ratios for LBW incidence. CI, confidence interval; LBW, low birth weight; OR, odds ratio.

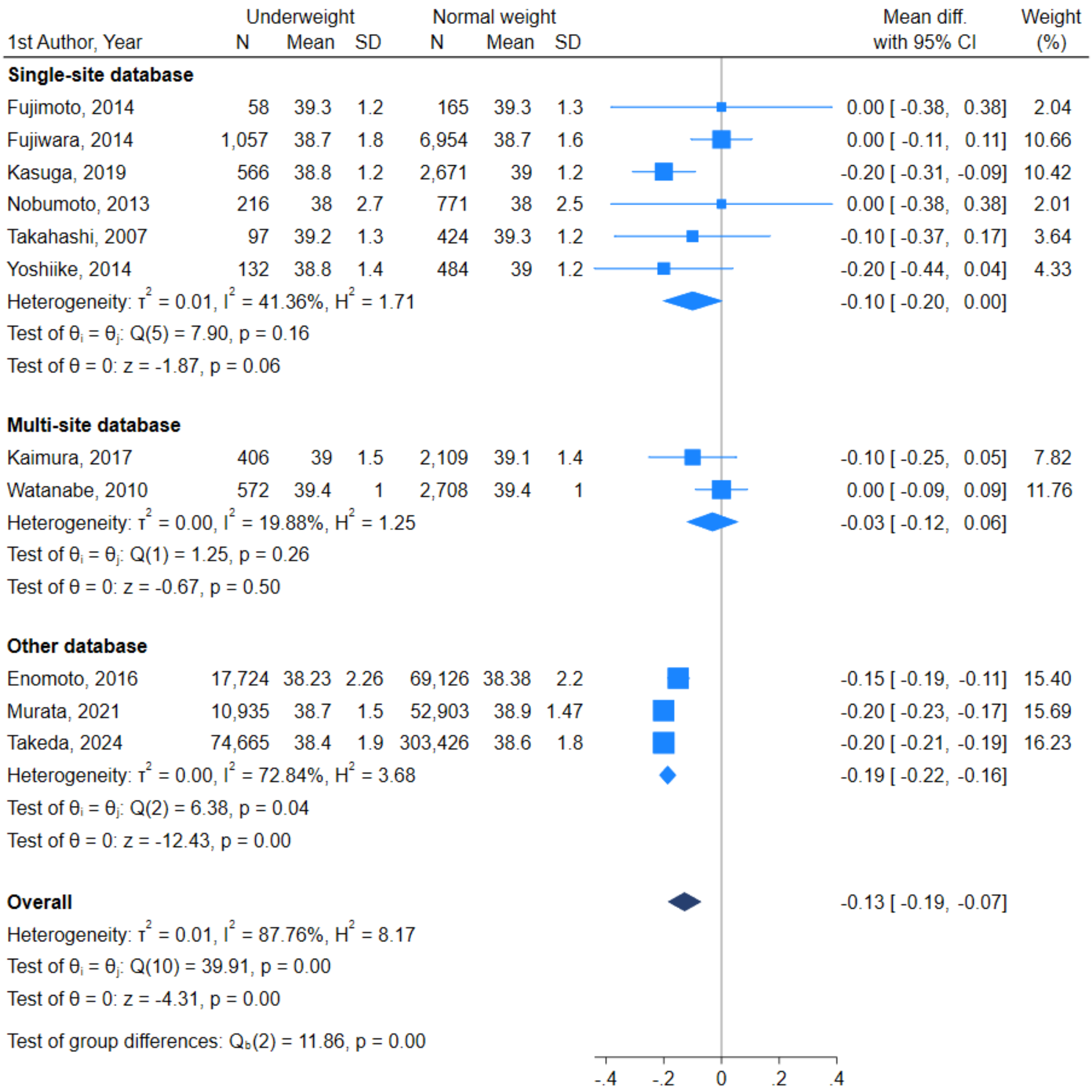


**eFigure 8.** Subgroup analysis: forest plot showing the association between pre-pregnancy underweight and preterm birth, Mean differences in gestational age at delivery between underweight and normal weight women. CI, confidence interval; OR, odds ratio; SD, standard deviation.

**eMaterial 1.** Search terms for MEDLINE

| Number | Searches | Results |
|---|---|---|
| 1 | exp Pregnancy/ | 1,024,821 |
| 2 | exp Pregnancy Complications/ | 481,526 |
| 3 | Pregnant Women/ | 15,587 |
| 4 | Preconception Care/ | 2,721 |
| 5 | 1 or 2 or 3 or 4 | 1,060,199 |
| 6 | Time-to-Pregnancy/ | 323 |
| 7 | exp Pregnancy Rate/ | 23,463 |
| 8 | Maternal Mortality/ | 11,487 |
| 9 | exp Reproductive History/ | 29,693 |
| 10 | 6 or 7 or 8 or 9 | 62,972 |
| 11 | pregnan*.ti,ab,kf. | 630,436 |
| 12 | (gravida* or maternit* or expectant mother* or gravidity or parity).ti,ab,kf. | 77,832 |
| 13 | (prepregnan* or preconception* or pre conception*).ti,ab,kf. | 12,858 |
| 14 | 11 or 12 or 13 | 679,646 |
| 15 | 5 or 10 or 14 | 1,232,067 |
| 16 | Body Mass Index/ | 151,560 |
| 17 | exp Thinness/ | 15,058 |
| 18 | exp Overweight/ | 279,983 |
| 19 | Body Weight/ | 200,588 |
| 20 | (BMI or body mass index).ti,ab,kf. | 335,278 |
| 21 | (underweight* or thinness or leanness or emaciation*).ti,ab,kf. | 21,352 |
| 22 | (overweight* or obesit* or obese or fatness or adipositas or adiposity or corpulen).ti,ab,kf. | 435,027 |
| 23 | or/16-22 | 877,356 |
| 24 | 15 and 23 | 65,608 |
| 25 | exp Japan/ | 157,742 |
| 26 | Japan*.ti,ab,kf. | 268,894 |
| 27 | Japan*.in. | 1,474,941 |
| 28 | 25 or 26 or 27 | 1,612,542 |
| 29 | 24 and 28 | 1,708 |
| 30 | Case Reports.pt. | 2,385,919 |
| 31 | (case adj1 (report? or study or studies or history or histories or series or presentation?)).ti. | 441,817 |
| 32 | 30 or 31 | 2,510,105 |
| 33 | 29 not 32 | 1,672 |
| 34 | exp animals/ not humans.sh. | 5,198,404 |
| 35 | 33 not 34 | 1,054 |

Note: Date of search: February 19, 2024.

**eMaterial 2.** Search terms for EMBASE

| Number | Searches | Results |
|---|---|---|
| #1 | 'pregnancy'/exp | 897,370 |
| #2 | 'pregnancy disorder'/exp | 770,706 |
| #3 | 'pregnant woman'/de | 117,187 |
| #4 | 'prepregnancy care'/de | 3,114 |
| #5 | #1 OR #2 OR #3 OR #4 | 1,341,875 |
| #6 | 'time to pregnancy'/de | 1,612 |
| #7 | 'pregnancy rate'/de OR 'birth rate'/de | 54,704 |
| #8 | 'maternal mortality'/de | 26,597 |
| #9 | 'reproductive history'/de OR 'parity'/de | 49,739 |
| #10 | #6 OR #7 OR #8 OR #9 | 128,945 |
| #11 | pregnan*:ti,ab,kw | 835,947 |
| #12 | gravid*:ti,ab,kw OR maternit*:ti,ab,kw OR 'expectant mother*':ti,ab,kw OR parity:ti,ab,kw | 109,676 |
| #13 | prepregnan*:ti,ab,kw OR preconception*:ti,ab,kw OR 'pre conception*':ti,ab,kw | 27,404 |
| #14 | #11 OR #12 OR #13 | 901,463 |
| #15 | #5 OR #10 OR #14 | 1,561,406 |
| #16 | 'body mass'/de | 660,357 |
| #17 | 'underweight'/de | 23,901 |
| #18 | 'obesity'/exp | 694,427 |
| #19 | 'body weight'/de OR 'ideal body weight'/de | 410,495 |
| #20 | bmi:ti,ab,kw OR 'body mass index':ti,ab,kw | 608,860 |
| #21 | underweight*:ti,ab,kw OR thinness:ti,ab,kw OR leanness:ti,ab,kw OR emaciation*:ti,ab,kw | 30,477 |
| #22 | overweight*:ti,ab,kw OR obesit*:ti,ab,kw OR obese:ti,ab,kw OR fatness:ti,ab,kw OR adipositas:ti,ab,kw OR adiposity:ti,ab,kw OR corpulent:ti,ab,kw | 647,195 |
| #23 | #16 OR #17 OR #18 OR #19 OR #20 OR #21 OR #22 | 1,634,050 |
| #24 | #15 AND #23 | 126,867 |
| #25 | 'japan'/de | 210,282 |
| #26 | 'japanese (people)'/de | 70,684 |
| #27 | japan:ca | 1,755,920 |
| #28 | japan*:ti,ab,kw | 439,654 |
| #29 | #25 OR #26 OR #27 OR #28 | 1,986,242 |
| #30 | #24 AND #29 | 3,227 |
| #31 | 'case report'/de OR 'case study'/exp | 3,120,353 |
| #32 | (case NEXT/1 (report* OR study OR studies OR history OR histories OR series OR presentation*)):ti | 557,574 |
| #33 | #31 OR #32 | 3,223,737 |
| #34 | #30 NOT #33 | 2,955 |
| #35 | ('animal'/exp OR 'animal experiment'/exp OR 'animal model'/exp OR 'nonhuman'/de) NOT ('human'/exp OR 'human experiment'/exp) | 7,948,591 |
| #36 | #34 NOT #35 | 2,117 |
| #37 | #36 AND ('Article'/it OR 'Article in Press'/it OR 'Review'/it) | 1,428 |

Note: Date of search: February 19, 2024.

**eMaterial 3.** Search terms for CINAHL

| Number | Searches | Results |
|---|---|---|
| S1 | (MH "Pregnancy+") | 244,072 |
| S2 | (MH "Pregnancy Complications+") | 110,204 |
| S3 | (MH "Expectant Mothers") | 14,496 |
| S4 | (MH "Prepregnancy Care") | 2,380 |
| S5 | S1 OR S2 OR S3 OR S4 | 264,764 |
| S6 | (MH "Maternal Mortality") | 7,165 |
| S7 | MH reproductive history OR MH Parity | 7,411 |
| S8 | S6 OR S7 | 14,461 |
| S9 | TX pregnan# | 64,453 |
| S10 | TX (gravida or maternity or "expectant mother" or gravidity or parity) | 41,896 |
| S11 | TX (prepregnan# or preconception or pre conception) | 3,748 |
| S12 | S9 OR S10 OR S11 | 103,318 |
| S13 | S5 OR S8 OR S12 | 302,753 |
| S14 | (MH "Body Mass Index") | 97,519 |
| S15 | (MH "Thinness") | 3,917 |
| S16 | (MH "Obesity+") | 115,779 |
| S17 | (MH "Body Weight") | 35,344 |
| S18 | TX (BMI or body mass index) | 157,502 |
| S19 | TX (underweight or thinness or leanness or emaciation) | 8,999 |
| S20 | TX (overweight or obesity or obese or fatness or adipositas or adiposity or corpulen) | 180,397 |
| S21 | S14 OR S15 OR S16 OR S17 OR S18 OR S19 OR S20 | 296,628 |
| S22 | S13 AND S21 | 20,540 |
| S23 | (MH "Japan") | 37,614 |
| S24 | (MH "Japanese Persons") | 2,608 |
| S25 | AF (Japan or Japanese) | 166,901 |
| S26 | TX (Japan or Japanese) | 194,052 |
| S27 | S23 OR S24 OR S25 OR S26 | 194,052 |
| S28 | S22 AND S27 | 372 |
| S29 | TI ("case report" or "case study" or "case series" or "case presentation") | 105,327 |
| S30 | S28 NOT S29 | 369 |
| S31 | (MH "Animals+") | 102,881 |
| S32 | (MH "Animal Studies") | 156,361 |
| S33 | TI animal model | 3,657 |
| S34 | S31 OR S32 OR S33 | 250,113 |
| S35 | (MH "Human") | 2,769,062 |
| S36 | S34 NOT S35 | 215,687 |
| S37 | S30 NOT S36 | 340 |

Note: Date of search: February 19, 2024.

**eMaterial 4.** Search terms for PsycINFO

| Number | Searches | Results |
|---|---|---|
| 1 | exp pregnancy/ | 50,460 |
| 2 | expectant mothers/ | 1,075 |
| 3 | pregnan*.mp. | 77,023 |
| 4 | (gravida* or maternit* or expectant mother* or gravidity or parity).mp. | 11,308 |
| 5 | (prepregnan* or preconception* or pre conception*).mp. | 3,027 |
| 6 | or/1-5 | 96,994 |
| 7 | body mass index/ | 8,466 |
| 8 | exp underweight/ | 13,267 |
| 9 | exp overweight/ | 31,172 |
| 10 | body weight/ or birth weight/ or weight control/ | 22,114 |
| 11 | (BMI or body mass index).mp. | 39,490 |
| 12 | (underweight* or thinness or leanness or emaciation*).mp. | 5,671 |
| 13 | (overweight* or obesit* or obese or fatness or adipositas or adiposity or corpulen).mp. | 57,376 |
| 14 | or/7-13 | 101,382 |
| 15 | 6 and 14 | 7,321 |
| 16 | Japan*.mp. | 45,224 |
| 17 | Japan*.in,lo. | 102,284 |
| 18 | 16 or 17 | 114,551 |
| 19 | 15 and 18 | 154 |

Note: Date of search: February 19, 2024.

**eMaterial 5.** Search terms for Cochrane Library (CENTRAL)

| Number | Searches | Results |
|---|---|---|
| #1 | MeSH descriptor: (Pregnancy) explode all trees | 33,699 |
| #2 | MeSH descriptor: (Pregnancy Complications) explode all trees | 17,593 |
| #3 | MeSH descriptor: (Pregnant Women) this term only | 988 |
| #4 | MeSH descriptor: (Preconception Care) this term only | 196 |
| #5 | #1 or #2 or #3 or #4 | 37,926 |
| #6 | MeSH descriptor: (Time-to-Pregnancy) this term only | 26 |
| #7 | MeSH descriptor: (Pregnancy Rate) explode all trees | 2,402 |
| #8 | MeSH descriptor: (Maternal Mortality) this term only | 199 |
| #9 | MeSH descriptor: (Reproductive History) explode all trees | 1,293 |
| #10 | #6 or #7 or #8 or #9 | 3,887 |
| #11 | pregnan*:ti,ab,kw | 87,532 |
| #12 | (gravida* or maternit* or expectant mother* or gravidity or parity):ti,ab,kw | 8,064 |
| #13 | (prepregnan* or preconception* or pre conception*):ti,ab,kw | 1,912 |
| #14 | #11 or #12 or #13 | 90,189 |
| #15 | #5 or #10 or #14 | 92,916 |
| #16 | MeSH descriptor: (Body Mass Index) this term only | 13,971 |
| #17 | MeSH descriptor: (Thinness) explode all trees | 845 |
| #18 | MeSH descriptor: (Overweight) explode all trees | 24,688 |
| #19 | MeSH descriptor: (Body Weight) this term only | 10,791 |
| #20 | (BMI or body mass index):ti,ab,kw | 90,081 |
| #21 | (underweight* or thinness or leanness or emaciation*):ti,ab,kw | 1,717 |
| #22 | (overweight* or obesit* or obese or fatness or adipositas or adiposity or corpulen):ti,ab,kw | 60,411 |
| #23 | #16 or #17 or #18 or #19 or #20 or #21 OR #22 | 128,248 |
| #24 | #15 and #23 | 10,540 |
| #25 | MeSH descriptor: (Japan) explode all trees | 5,061 |
| #26 | (Japan*) (Word variations have been searched) | 53,434 |
| #27 | #25 or #26 | 53,444 |
| #28 | #24 and #27 | 107 |
| #29 | (Conference proceeding):pt | 236,547 |
| #30 | #28 not #29 | 77 |

Note: Date of search: February 19, 2024.

**eMaterial 6.** Search terms for *Ichushi*

| Num ber | Searches | Results |
|---|---|---|
| #1 | "*ninshin*"/TH | 74,997 |
| #2 | "*ninsanpu*"/TH | 11,300 |
| #3 | "*ninshin-gappeisho*"/TH | 162,122 |
| #4 | "*ninshinmaekanri*"/TH | 1,108 |
| #5 | CK=*ninshin* | 199,458 |
| #6 | #1 or #2 or #3 or #4 or #5 | 267,923 |
| #7 | "*ninshin-madeno-kikan*"/TH | 13 |
| #8 | "*seisyokuk-reki"*/TH | 4,027 |
| #9 | "*ninshin-ritsu*"/TH | 7,142 |
| #10 | "*ninnsanpu-shibouritsu*"/TH | 480 |
| #11 | "*ninsanpu-shibou*"/TH | 998 |
| #12 | #7 or #8 or #9 or #10 or #11 | 12,288 |
| #13 | *ninshin*/TA | 155,146 |
| #14 | *ninpu* or/TA and *ninsanpu*/TA or *sanpu* /TA or *sanjyokuhu* /TA | 50,816 |
| #15 | pregnan/TA or gravid/TA or maternit/TA or parity/TA | 7,973 |
| #16 | *pre-konsepushon* /TA or preconception/TA or "pre-conception"/TA | 601 |
| #17 | #13 or #14 or #15 or #16 | 199,068 |
| #18 | #6 or #12 or #17 | 349,692 |
| #19 | "BMI"/TH | 26,357 |
| #20 | BMI/TA or "body mass index"/TA | 26,023 |
| #21 | *himan-shisu* /TA or *taikaku-shisu* /TA or *taijyu-shisu* /TA | 1,761 |
| #22 | #19 or #20 or #21 | 41,718 |
| #23 | "*yase*"/TH | 3,776 |
| #24 | *yase* /TA or *yase (kanji)* /TA or *teitaijyu* /TA or *ruisou* /TA or *ruisou (kanji)* /TA | 9,800 |
| #25 | underweight/TA or thinness/TA or leanness/TA or emaciation/TA | 98 |
| #26 | #23 or #24 or #25 | 11,403 |
| #27 | "*himan*"/TH | 73,042 |
| #28 | *kajyotaijyu* /TA or *kataijyu* /TA or *taijyu*-ka /TA or *himan* /TA | 75,762 |
| #29 | overweight/TA or obesit/TA or obese/TA or fatness/TA or adipositas/TA or adiposity/TA or corpulent/TA | 4,887 |
| #30 | #27 or #28 or #29 | 99,694 |
| #31 | #22 or #26 or #30 | 135,957 |
| #32 | #18 and #31 | 5,447 |
| #33 | (#32) and (PT=original article) | 2,281 |
| #34 | (#32) and (PT=review) | 127 |
| #35 | #33 or #34 | 2,408 |
| #36 | (#35) and (PT=case report excluded) | 2,049 |
| #37 | CK=*doubutsu* | 1,054,152 |
| #38 | CK=*hito* | 9,508,580 |

| | | |
|---|---|---|
| #39 | #37 not #38 | 925,853 |
| #40 | #36 not #39 | 1,950 |

Note: Date of search: February 19, 2024. For the *Ichushi* database, searches were conducted using Japanese terms. The italicized terms shown in this table are the Romanized (Roman alphabet) representations of the original Japanese search terms.
